# *Drosophila* Jak/STAT Signaling: Regulation and Relevance in Human Cancer and Metastasis

**DOI:** 10.3390/ijms19124056

**Published:** 2018-12-14

**Authors:** Sunny Trivedi, Michelle Starz-Gaiano

**Affiliations:** Department of Biological Sciences, University of Maryland Baltimore County, 1000 Hilltop Circle, Baltimore, MD 21250, USA; sunny4@umbc.edu

**Keywords:** Janus kinase (Jak) 1, signal transducer and activator of transcription (STAT) 2, *Drosophila* 3, cancer metastasis 4.

## Abstract

Over the past three-decades, Janus kinase (Jak) and signal transducer and activator of transcription (STAT) signaling has emerged as a paradigm to understand the involvement of signal transduction in development and disease pathology. At the molecular level, cytokines and interleukins steer Jak/STAT signaling to transcriptional regulation of target genes, which are involved in cell differentiation, migration, and proliferation. Jak/STAT signaling is involved in various types of blood cell disorders and cancers in humans, and its activation is associated with carcinomas that are more invasive or likely to become metastatic. Despite immense information regarding Jak/STAT regulation, the signaling network has numerous missing links, which is slowing the progress towards developing drug therapies. In mammals, many components act in this cascade, with substantial cross-talk with other signaling pathways. In *Drosophila*, there are fewer pathway components, which has enabled significant discoveries regarding well-conserved regulatory mechanisms. Work across species illustrates the relevance of these regulators in humans. In this review, we showcase fundamental Jak/STAT regulation mechanisms in blood cells, stem cells, and cell motility. We examine the functional relevance of key conserved regulators from *Drosophila* to human cancer stem cells and metastasis. Finally, we spotlight less characterized regulators of *Drosophila* Jak/STAT signaling, which stand as promising candidates to be investigated in cancer biology. These comparisons illustrate the value of using *Drosophila* as a model for uncovering the roles of Jak/STAT signaling and the molecular means by which the pathway is controlled.

## 1. Introduction

The Janus kinase (Jak) and Signal transducer and activator of transcription (STAT) signaling pathway is crucial in the regulation of the immune response, in stem cell regulation, and in determining cell identities in diverse organisms. In the late 1980s and early 1990s, this signaling cascade was shown to be central to the interferon response in humans (reviewed in [1,2]), and its homologs were soon identified in *Drosophila* [3,4,5,6,7]. The demonstration that activating mutations in Jak produced neoplastic growth in flies, particularly in blood cell-like lineages [8,9,10,11], illustrated the striking similarity between the pathways across the animal kingdom, because, soon after, deregulated STAT function was linked to human hematopoietic malignancies and activating mutations in Jak were linked to leukemia and other myeloproliferative disorders [1,12,13,14]. Given these parallels, it is no surprise that detailed characterization of the Jak/STAT pathway in *Drosophila* has been very informative about its functional mechanisms in humans.

Here, we broadly compare and contrast the Jak/STAT signaling cascade in mammals and *Drosophila*. We review studies linking key Jak/STAT activity regulators with human disease, especially blood cell cancer, cancer stem cells, and metastatic cancers derived from breast and prostate. We also describe well-characterized cell types and phenotypes affected by loss and gain of function of Jak/STAT pathway components in *Drosophila*, and discuss how flies can be useful for the identification of new pathway regulators. Finally, we explore connections between genes determined to be Jak/STAT regulators in *Drosophila* and their human homologs that are linked to disease, and highlight candidates for further study based on their involvement in both contexts.

## 2. Jak/STAT Signaling Overview in Flies and Humans

**Figure 1:**
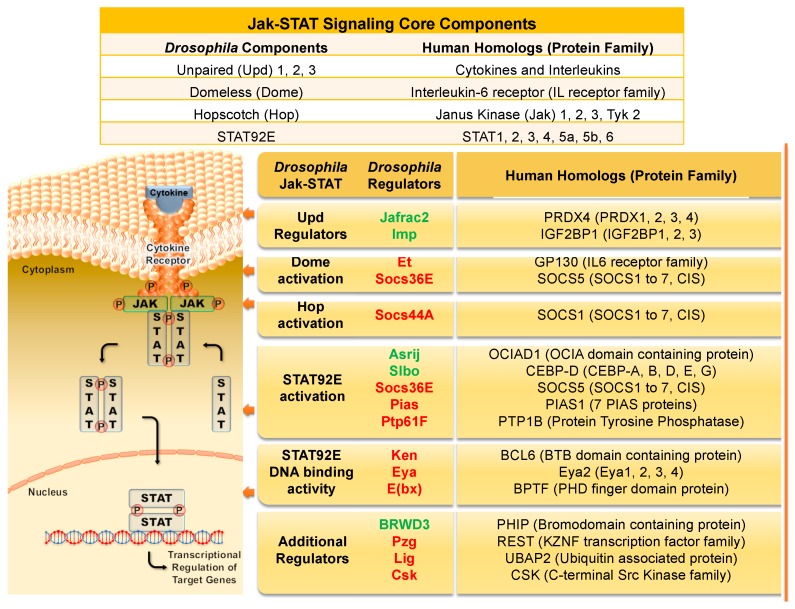
The *Drosophila* Jak/STAT signaling components and the corresponding human homologs and their protein families. Interleukin or cytokine (the Upd family in flies) binds to its signaling receptor (Dome in flies), which activates the associated Jak (fly Hop) and triggers a chain of events. Activated Jak phosphorylates other Jaks and the receptor, creating a binding site for STAT proteins. Recruited STAT proteins (fly STAT92E), are then phosphorylated. The phospho-STATs dimerize and translocate to the nucleus. The STAT DNA binding domain recognizes promoter and enhancer regions of target genes, resulting in their transcriptional activation. The table above the figure lists the core components of the canonical pathway. The table to the right delineates key regulators of the fly Jak/STAT pathway, their respective human homologs, and their protein families. Positive regulators are listed in green font, negative regulators are in red. See text for details.

Extracellular cues trigger Jak/STAT signaling, which ultimately leads to transcriptional activation of target genes (Figure 1). The basic framework for this signaling is the same across species, but the mammalian signaling system includes families of proteins with overlapping roles, whereas the fly cascade has fewer components and less redundancy. In humans, a set of more than 40 interleukins and cytokines serve as activating cues (reviewed in [15,16,17]). In flies, only three proteins hold this function: Unpaired (Upd) 1, Upd 2, and Upd 3 [4,18,19,20]. Given the array of activators, mammals have multiple cell-surface receptors that can act singly or multimerize to respond to their diverse set of ligands [21,22]. In contrast, one signaling receptor has been determined in flies, called Domeless (Dome) [5,6,23,24], which can interact with the non-signaling receptor, Eye transformer (Et, similar to human type I receptor GP130) [25,26,27]. Receptor–ligand binding activates Jak proteins docked to the cytoplasmic portion of the receptors. There are four Janus kinases in humans (Jak1–3 and Tyrosine kinase 2 (Tyk2)), which bind different receptors. One Jak protein is found in flies, which is most similar to human Jak 2. Like most *Drosophila* genes, the gene encoding Jak is named after its loss of function phenotype; due to defective segmentation and skipped segments in the cuticular patterns of late embryos and early larvae, the mutant was named *hopscotch* (*hop*, with similar phenotypes observed for *unpaired* mutants) [10,28]. Janus kinases have a well-conserved structure, featuring a kinase domain, a similar pseudokinase domain without catalytic activity, and a band 4.1- ezrin-radixin-moesin (FERM) domain that binds to the receptor and contributes to the regulation of kinase activation upon receptor–ligand binding [29]. Activated Jak targets a second Jak associated within the same receptor dimer or multimer, and the subsequent phosphorylations create binding sites for cytoplasmic STAT proteins. There exist seven STAT family members in humans (STAT1–4, 5a, 5b, and 6), but only one in flies: STAT92E, which is most similar to STAT5b [3,7,30]. Conserved domains in STAT proteins include the coiled coil, Src Homology 2 (SH2), DNA binding, and transactivation domains [21]. Non-phosphorylated STATs have been shown to have several functions in flies, including promoting heterochromatin formation with HP1 and maintaining genomic stability [31,32,33]. Likewise, some STAT family members can function in mammalian cells without being phosphorylated, for example by interacting with cytoskeletal regulators, functioning at mitochondria or Golgi, modulating NF-κB signaling, increasing heterochromatin, or heterodimerizing with phosphorylated STATs [31,34,35]. However, the best studied roles for the protein family are those that occur after it is "activated" by phosphorylation. Phosphorylated STATs dimerize, which promotes their translocation into the nucleus where they directly bind DNA and recruit transcriptional activators [21]. Thus, the canonical Jak/STAT pathway results in changes in gene expression, including amplifying expression of its own regulators. 

As the various STAT proteins in humans can homo- or hetero-dimerize, and can be activated by numerous permutations of the ligands, receptors, and Jaks, the combinatorial outcomes are very complex. Thus, the stripped-down pathway that exists in *Drosophila* is important to provide a tractable, but still very relevant, system for characterization of this signaling cascade and its essential regulators.

### 2.1. Requirement of Jak/STAT Signaling in Development and Adulthood

In both flies and mammals, normal early development requires correct Jak/STAT signaling, and spatiotemporal pathway misregulation later in life is detrimental. Humans with inborn errors in Jak/STAT genes that are important in blood cell lineages are immunocompromised [36]. Additionally, abnormally high Jak and STAT activities in adults have been closely associated with autoimmune diseases, cell overproliferation, acquisition of blood cell disorders, cancer progression, and poor cancer prognosis [14,15,37,38,39]. In light of this, much research is directed at understanding this signaling pathway.

While some null Jak/STAT pathway mutations cause tissue-specific defects, presumably others would not allow human development to term, as evidenced by mouse genetic studies. Mutations in genes encoding positive signaling components result in early lethality in mice or cell type-specific effects [21,40]. For example, mutant *Jak1* and *Jak3* mice have severe combined immunodeficiency (SCID), and *Jak1* mutants also have neurological defects and poor survival past birth; knock out mutations in *Jak2* are embryonic lethal, and mutations in the Jak family member *Tyk2* result in poor response to pathogens [29]. Similarly, *Stat1* mutant mice have abnormal immune responses and are more susceptible to infections than wild type [40], and show significant neurodegeneration as adults [41]. *Stat3* mutant mice die in early embryogenesis and tissue-specific mutations result in changes in the proliferation–apoptosis balance in blood cells, poorer cell motility, and inflammation [21,40]. *Stat5a* and *b* have overlapping, required roles in mammary gland and ovary development, as well as being important in blood cell proliferation and cytotoxic activity [40,42]. Female *Stat5a/b* double knock out mice are sterile.

Mutations that block Jak/STAT signaling in *Drosophila* result in early lethality; however, this can be overcome experimentally using sophisticated genetic tools that allow fly researchers to test mutations in individual cell types or at certain times in development. These types of experiments can be performed by using temperature sensitive mutations, by tissue-specific expression control through the Gal4/UAS system, or by using clonal mosaic analysis, in which most cells of the organism are heterozygous and small clones of cells are homozygous mutant. (For reviews on methodology and Jak/STAT related genetic tools, see [43,44]). These strategies revealed essential functions for Jak/STAT signaling in sex determination [45,46,47,48], as well as in cellular functions in diverse cell types that include fly blood cells [49,50], wing precursors [51,52], eye progenitor cells [53], gut stem cells [54,55,56], adult testes stem cells [57,58,59], and adult ovary cell types [23,24,60,61] (see Table 1). (For recent reviews on *Drosophila* Jak/STAT signaling in specific contexts, see [62,63,64,65] (stem cells), [66] (tumors), [49,50,67] (hematopoiesis and immunity), [68] (morphogenesis), [31] (heterochromatin), [69] (cell–cell competition), and [70] (host–parasite interactions)). In many of these contexts, loss of signaling produced abnormal phenotypes, as did unusually high levels of signaling. We discuss several specific cases below. These experiments implicate Jak/STAT functional disruptions in defects in stem cell maintenance, cell survival, proliferative defects, cell fate specification, and cell migration in a variety of tissue types.

### 2.2. Jak/STAT Activity Regulators

Given the many diverse roles for Jak/STAT signaling, and the fact that either too much or too little signaling can produce abnormal effects, it is not surprising that the pathway is subject to many levels of regulation. Estimates based on *Drosophila* cell culture screens suggest there are on the order of hundreds of regulators [71,72,73].

Multiple regulatory proteins were initially discovered in mammalian contexts, then shown to play similar roles in flies. Among the first to be characterized was the family of proteins called Protein inhibitor of activated STAT (PIAS). These proteins bind to activated STATs to block DNA binding and transcriptional activity, and have roles in the sumoylation and downregulation and degradation of signaling components [74,75,76,77]. There are seven mammalian PIAS proteins encoded by four genes, and each can differentially target STATs or affect other transcription factors. The single PIAS encoded in *Drosophila* inhibits STAT activity in blood cells and in the eye [78]. Members of the suppressor of cytokine signaling (SOCS) pathway function as feedback inhibitors of STAT activity in mammals and flies [21,79]. These can bind Jaks or the receptor directly through an SH2 domain and reduce kinase activity, which occurs through recruitment of proteins to promote ubiquitination and degradation [80,81]. Eight SOCS proteins have been characterized in mammals; several of these have specific regulatory links to certain STATs, as well as roles in down-regulating other signaling pathways. Three SOCS proteins are encoded in flies but only two (Socs36E and Socs44A) are known to regulate Jak/Stat signaling [82,83,84] and, in some contexts, these also modulate signaling through the epidermal growth factor receptor (EGFR), Ras, and Mitogen activated protein (MAP) kinases [85,86]. When EGFR is activated, Socs36E functions as a tumor suppressor in epithelia and, similarly, depletion of SOCS5 function has a synergistic effect on cell transformation when combined with activation of EGFR or Ras/MAP kinase signaling in human cell culture [87]. Fly Socs36E has an intrinsically disordered domain and can recruit upstream Jak/STAT pathway components to Cullin-dependent degradation [88], similar to how it acts in human cells. In both flies and mammals, STAT transactivates *Socs* gene expression, creating feedback inhibition of the pathway [79,83,89,90,91,92]. An additional well-characterized class of regulatory proteins is the protein tyrosine phosphatases (PTPs), which directly dephosphorylate Jaks and potentially STATs [71,93,94], reversing their activation. This is a very large family in mammals with cell type-specific expression; multiple members have been shown to function in Jak/STAT signaling, especially in cell type-specific ways [95]. So far, one member of this family, Ptp61F, is linked to Jak/STAT signaling in *Drosophila* [71,73,96]. Another negative regulator is the transmembrane receptor, Et, which, although it is structurally similar to mammalian GP130, acts as an inhibitory receptor by interacting with Dome and blocking its homo-dimerization and activation [25,26,27]. Finally, regulation of receptor internalization via endocytosis is a regulatory mechanism common across species that modulates Jak/STAT signaling [97,98,99,100,101,102].

More recently, flies have been used to identify novel regulators of Jak/STAT signaling. This has been accomplished by identifying mutants with similar phenotypes, by leveraging unbiased genetic enhancement and suppression screening, and by assaying pathway activity in cell culture. Many of the proteins identified in this way have human homologs that may play analogous roles. Given that Jak/STAT activity has important functions in immune response, cell motility, and stem cell maintenance in both humans and flies, genetic analysis in the fly provides a valuable strategy to elucidate critical regulators in these processes. Thus, studying different cellular contexts and using multiple approaches will be useful for determining how these components are controlled and how they normally fine-tune the pathway to prevent pathological states. We will describe Jak/STAT regulation in three contexts: Mammalian blood cell differentiation and proliferation, stem cell signaling, and cell motility and metastasis. Next, we will draw parallels with regulation of these processes in flies via Jak/STAT signaling. Finally, we will explore the potential contributions for homologs of *Drosophila* Jak/STAT regulators in metastasis.

## 3. Jak/STAT Signaling in Blood Cell Proliferation and Cell Fate

### 3.1. Jak/STAT Signaling and Human Blood Cell Development and Disease

In humans, blood cells are produced from hematopoietic stem cells (HSC), which reside in a stem cell niche in bone marrow. HSCs divide asymmetrically to renew and make multipotent daughter cells; depending on local factors, these will produce multipotent stem cells restricted either to lymphoid or myeloid lineages. Jak/STAT signaling is important in both stem cell regulation and differentiation. STAT5 is expressed in HSCs and is necessary for their ability to self-renew [39,103], and STAT3 is also active in HSCs but is not strictly required [104]. Mouse knock out experiments demonstrate that both STAT3 and STAT5 are needed for B-cell development, and STAT5 is necessary for differentiation of some myeloid cell types [39].

Multiple different mutations in the human Jak/STAT signaling pathway or its positive regulators result in a defective immune response or proliferative disorders in blood cells [14,21,37,39]. Loss of function mutations are linked to immune diseases and the inability to fight certain kinds of infections [14,36]. Conversely, aberrantly high Jak/STAT signaling is well-known for promoting myeloproliferative disorders, including leukemia and lymphoma [14,39]. In these diseases, extra cell division and poor differentiation of certain white blood cell types in bone marrow renders the immune system dysfunctional and causes overproliferation and tumors. Genetic fusions between Jak2 and the Translocation-Ets-leukemia (Tel/ETV6) gene that constitutively activate Jak appear to be causative for some cases of chronic myeloid leukemias and acute lymphoblastic leukemia. Other hematopoietic malignancies show activation of Jak1 or Jak3 through different means [14,39]. A rare chronic leukemia called myelofibrosis is most commonly caused by activating mutations in Jak2 [105]. This blood cell disorder alters stem cell dynamics in bone marrow and results in scarring or fibrosis of the bone marrow, disrupting the stem cell niche. Similarly, polycythemia vera is a rare neoplastic blood disorder often caused by activating mutations in Jak2 that result in the overproduction of red blood cells [106]. Transformation of blood cells by oncogenes often results in aberrant activation of STAT1, 3, or 5, depending on the originating cell type, and this can occur independently from canonical upstream signaling [14,39]. This activation can promote proliferation, differentiation defects, and stem cell-like character or neoplasia.

Since activating mutations lead to disease, several drugs are currently used therapeutically to suppress Jak/STAT signaling in patients. AG490 was the first Jak inhibitor characterized, and it has been used in the treatment of acute lymphoblastic leukemia; other drug analogs have been shown to be effective in blocking acute myeloid leukemia progression and survival in cell culture [14,39,107,108]. A Jak1/2 inhibitor, ruxolitinib (Jakifi), is the only therapeutic agent for the treatment of myelofibrosis in the US. Tofacitinib (Xeljanz), a pan-Jak inhibitor, modulates the immune response via Jak1/3 and STAT1 [109], and is approved in the US for treatment of inflammatory arthritis and rheumatoid arthritis. The Jak inhibitor, ADZ1480, has been found to be effective in blocking the growth and survival of cell lines derived from multiple different solid tumors and carcinomas [39]. While these drugs are promising and more clinical trials are underway, many have significant side effects, indicating that better drugs are needed.

### 3.2. Drosophila Hemocytes as a Model for Blood Disorders

*Drosophila* hemocytes show many similarities to human white blood cells and play critical roles in the immune response. The fly innate immune system acts through the production of antimicrobial peptides and the phagocytosis of pathogens. *Drosophila* have an open circulatory system and do not require oxygenated red blood cells. However, three types of blood cells differentiate from hemocytes: Plasmatocytes, which phagocytose bacteria and are most similar to human macrophages; crystal cells, which are responsible for melanization of pathogens; and lamellocytes, which encapsulate large foreign invaders like wasp eggs [67,70,110]. The main hematopoietic stem cells reside in the larval lymph gland. The posterior signaling center of the lymph gland produces Upd family members, which induces Jak/STAT signaling in hemocyte progenitors, maintaining the stem-like character and preventing them from differentiating too soon [49,111]. An interesting, recent study showed that proper blood cell differentiation decisions in the lymph gland depend on signaling from embryonic hemocytes [112]. Hemocytes express the transcription factor Glial cells missing (Gcm), which is required to activate Jak/STAT negative regulators and prevent the pro-inflammatory effects of overactivation Upd 2 and Upd 3 expression or infection. This indicates that initial waves of signaling control have repercussions much later in development.

Like in mammals, Jak/STAT interacts with multiple signaling pathways in the hematopoietic compartment to control hemocyte differentiation and proliferation. Interactors include the combined homolog of platelet-derived growth factor and vascular endothelial growth factor, PVF2 [113,114]; the Hippo pathway transcription factor, Yorkie [115]; the homolog of early B-cell factor, Collier [111]; and the GATA factors, U-shaped and Pannier, which promote prohemocytes and differentiation, respectively [116,117,118]. The Jak/STAT signaling regulator, Asrij, is expressed in blood cells and other cell types, suggesting it is important in differentiation as well [119], and it regulates endocytic turnover of signaling components [120]. Interestingly, many of the transcriptional targets of STAT are conserved between fly hemocyte-derived tumors and HeLa cells [121], again supporting the idea that the signaling pathway is similar. Recent work indicates that quiescent hemocyte stem cells reside in adult flies, in addition to larval lymph glands, and are activated in response to infection [122], but the effects of Jak/STAT signaling on this have not yet been revealed. However, since Jak/STAT signaling is activated upon immune challenge in *Drosophila*, the pathway is necessary for an effective immune response in larva and adults [49,50,67,123,124].

A dominant, activating mutation in *Drosophila* Jak, Hop^Tumorous-lethal^ (Hop^Tum^), has provided an invaluable tool in the study of Jak/STAT signaling in flies [10]. This point mutation creates a single glycine to glutamic acid change in the pseudokinase domain, which renders the catalytic domain constitutively active [8,9,10,11]. Mutants die in larval stages with dark, neoplastic tumors of a melanotic blood cell subtype, reminiscent of human leukemia [125]. A different point mutation, Hop^T42^, creates an activating amino acid substitution in the kinase-like domain, which results in phenotypes similar to those due to Hop^Tum^ expression [9]. When this conserved amino acid is substituted in mouse Jak2, the mammalian protein, likewise, is constitutively activated. Interestingly, expression of a causative fusion protein in acute myeloid leukemia, Runt-related transcription factor 1(Runx1/AML1)-ETO, also caused expansion of hemocyte precursors [126], supporting the idea that *Drosophila* hemocytes provide a reasonable model for leukemia mechanisms.

A number of drug screens for Jak/STAT inhibitors have been performed using a cultured cell line derived from *Drosophila* embryonic hemocytes [127,128,129,130]. Notably, drugs suppressing Jak activity in humans also work against the fly counterpart, indicating functional and structural conservation across species. For example, the commonly-used drug, ruxolitinib, inhibits Jak/STAT signaling in fly cell culture [130]. Notably, this line of work showed that methotrexate specifically inhibits Jak/STAT signaling as well as ruxolitinib does [129]. Transferring this finding to cultured Hodgkin lymphoma cells, the study showed that methotrexate can suppress signaling even in the presence of the V617F activating mutation in Jak. Additionally, a novel compound, MS1040, was identified as a specific Jak inhibitor in fly cells and mammals [131], which could have clinical relevance. In another interesting line of work, approved chemotherapeutic agents for humans were administered to flies bearing a genetically-induced intestinal stem cell tumor [132]. Although some drugs could reduce the tumors, one class additionally caused overproliferation of normal stem cells. This effect was mediated via Jak/STAT activation, and may reveal clues to the biology of tumor recurrence in humans. Since combination therapy is a strong direction for future treatments of myeloproliferative disorders and cancers broadly, more work to identify specific and effective drugs is needed, and Jaks are a good target. These studies illustrate the utility of drug screening in *Drosophila*.

## 4. Jak/STAT Regulation of Stem Cell Character

### 4.1. Jak/STAT Signaling, Carcinomas, and Cancer Stem Cells

Besides its involvement in blood cell cancers, Jak/STAT signaling activation is common in carcinomas. Activated STAT3 and STAT5, in particular, promote tumorigenesis and cancer stem cells, and this has been extensively reviewed [15,21,133,134,135,136]. Cancer stem cells are rare, but can proliferate and are believed to contribute to resistance to treatment and cancer recurrence. STAT activity inhibition slows cancer cell growth and may change stem-like character; therefore, drugs to block the pathway, like ADZ1480, are being heavily investigated. One model posits that the cancer recurrence common with STAT-positive cancers is due to the ability of STAT to provide signals that induce niche-like properties, maintaining cancer stem cells non-autonomously [135]. As with hematopoietic stem cells, cancer stem cells are subject to many regulatory factors that impact the decisions to self-renew or differentiate. Since these stem cells are rare and usually hard to identify, it is helpful to examine stem cell regulation in other contexts. Research of *Drosophila* has provided strong insight into the molecular regulation of stem cells and the niches that support them. 

### 4.2. Drosophila Testes Stem Cells

The *Drosophila* testis is an outstanding model for examining stem cell regulation, as it is well-characterized, clearly organized, and genetically accessible. Adult testes have two stem cell populations, the germline cells and the somatic cyst stem cells [65,137]. Distal hub cells and cyst stem cells both act as a stem cell niche for the germline stem cells to maintain pluripotency. As cells divide and move away from the niche, they begin to differentiate. Thus, the testis provides a well-suited context to study how different signals act to influence the balance of stem cells. As in blood cells, gain or loss of Jak/STAT signaling in testes results in dramatic phenotypes, disrupting stem cell regulation and proliferative control. In this case, low STAT activity leads to a loss of somatic stem cells, which in turn causes germ line stem cell loss due to differentiation [57,58,59,138,139]. Conversely, somatic overactivation of the pathway leads to excessive cyst stem cells, which expands the stem cell niche and leads to the production of higher numbers of germline stem cells. In somatic stem cells, the transcription factor, Zinc finger homeodomain 1 (Zfh1), is the key effector of Jak/STAT signaling, and it itself is necessary and sufficient to promote stem cell fate [57,140]. The Jak/STAT negative regulators, Ptp61F and Socs36E, are important in this tissue as well [85,138,139,141]. Higher Ptp61F resulted in more differentiated cells [141]. Loss of *Socs36E* led to excessive signaling, overproliferation of stem cells, and changes in stem cell adherence to the niche [139], which enables mutant cells to outcompete wild type neighbors [85]. While these phenotypes are due in part to changes in Jak/STAT signaling, these regulators also have other targets. For example, high Socs36E in testes downregulated MAP kinase signaling in addition to Jak/STAT activity [85].

Key transcription factors and epigenetic regulators have been found to impact the output of Jak/STAT activity in testes, and provide feedback on signaling to prevent it from getting too high or too low. The B-cell lymphoma Bcl6 homolog, Ken and Barbie (Ken), is needed in the somatic stem cells to repress the Jak/STAT inhibitor Ptp61F; thus, Ken indirectly maintains high enough levels of Jak/STAT activity for stem cell self-renewal [141]. Ken opposes STAT activity by repressing some STAT target genes. The positive STAT regulator nucleosome-remodeling factor (NURF) acts to promote Jak/STAT-mediated stem cell maintenance and prevents differentiation [142]. Conversely, the enhancer of polycomb, a part of a histone acetyltransferase complex [143,144], and the ubiquitously transcribed tetratricopeptide repeat gene on the X chromosome (dUTX), a histone demethylase [145], both increase *Socs36E* expression and downregulate STAT activity. These function to counteract Jak/STAT signaling and promote somatic cell differentiation instead of stem cell maintenance. Similarly, the transcription factor, Apontic, suppresses Jak/STAT signaling to limit the number of somatic stem cells and thus limit the size of the stem cell niche, also by promoting expression of *Socs36E* and probably a STAT-directed microRNA [146,147]. These results implicate chromatin regulation in STAT target gene expression, and illustrate the tight regulation required on the pathway to make the amount of signaling optimal.

## 5. Jak/STAT Signaling Promotes Cell Motility and Metastasis

Jak/STAT signaling activation has been linked to more metastatic cancers for a number of tumor types (Table 1). A number of lines of evidence support this idea, from higher levels of signal detected in human metastases to xenograph and cell culture assays, which show that carcinoma cells are more invasive or motile in response to higher Jak/STAT activity, especially that of STAT3 and STAT5 [14,21,37,39]. To draw comparisons to the fly model, we will focus on two well-studied cases, breast and prostate cancers, below. We focus our discussion to recent results linking Jak/STAT signaling to the acquisition of cell motility. It is worth noting that metastatic disease can also be promoted by immune system activation, which can be Jak/STAT dependent, but we will concentrate on cell-autonomous means of promoting motility. We go on to describe how Jak/STAT signaling and its regulation is critical in the proper determination of a motile cell type in *Drosophila*—the border cells —and we outline how border cell behaviors capture aspects of metastatic cell migration.

### 5.1. Jak/STAT Signaling in Breast Cancer Metastasis

About 80% of diagnosed breast cancers are invasive, which threatens advancement to metastatic disease [148]. Metastatic cancer is much more likely to be lethal than carcinoma in situ. Jak/STAT signaling is a key regulator of cell migration and proliferation in this context [14,21,37,39]. In particular, Jak2-STAT5b signaling is often over-activated during tumor cell proliferation and metastatic spread of breast cancer. An analysis of multiple breast cancer cell lines showed that STAT5b is often constitutively phosphorylated at Y699 and activated [149]. Invasion into Matrigel is commonly used to assess a cell type’s metastatic potential. STAT5b silencing significantly inhibited invasion of a metastatic breast cancer cell line (T47D), compared to controls. Further studies showed that combinational drug therapy targeting Jak2-STAT5b signaling inhibited breast cancer metastasis [150]. Drug treatment downregulated STAT5b nuclear localization, binding activity, and downstream target gene expression. Downregulation of Jak2-STAT3 by overexpression of WW domain-containing oxidoreductase (Wwox), which inhibits Jak2 phosphorylation, attenuated cell migration in vitro and suppressed metastasis in vivo [151]. These data suggest Jak2-STAT5b significantly participate in promoting metastasis. A particularly hard-to-treat subtype of breast cancer is triple-negative breast cancer (TNBC), which lacks the human epidermal growth factor receptor 2 (HER2) and hormone receptors for estrogen and progesterone [152]. Jak2 is often amplified in TNBC cell lines and specific inhibition of Jak2-STAT5 signaling with the drug ruxolitinib reduced proliferation of cells in culture, as well as tumor growth in vivo [153], suggesting this is a good avenue for developing treatment regimes. Upstream of Jak2 and STAT3, IL6 is also linked to breast cancer metastasis: IL6 expression was found to be higher at the invasive leading edge of human primary breast cancer cells [154]. Overexpression of IL6 signaling induced metastasis and tumor growth in mouse models, while its downregulation suppressed growth and reduced metastatic progression. Thus, the core components of canonical Jak/STAT signaling seem to be integral in mediating cell proliferation and metastatic disease.

Layered over the core signaling, conserved regulators of Jak/STAT signaling also impact human breast cancer metastasis, although not always as expected. Given the key role of Jak2, it is clear that regulators mediating its degradation have a critical function in preventing overactivation. SOCS protein family members are known for their negative regulation of Jak/STAT signaling [81,155]. SOCS1 is overexpressed in TNBC tissues and cell lines [156]. SOCS1 is significantly associated with distant metastasis and its downregulation suppressed the proliferation of TNBC. Overexpression of SOCS1 protein correlates with lymph node metastasis, large tumor size, and advanced clinical stages in TNBC patients, but it is not likely to be targeting Jak/STAT in this case. In contrast, SOCS3 promotes ubiquitination of Jak2 to reduce its expression and regulate cytokine signaling, whereas tropomyosin-related kinase C (TrkC) prevents the SOCS3-mediated ubiquitination of Jak2 [157]. TrkC first binds and interacts with the c-Src/Jak2 complex to increase Jak2 and STAT3 levels, which induces Twist 1 and 2 expression. Carcinomas that become metastatic are thought to undergo an epithelial–mesenchymal transition (EMT), in which they lose apical basal polarity, reduce E-cadherin-based adhesions, and adopt the characteristics of migratory, loosely-connected mesenchymal cells. Twist 1 and 2 are known to be EMT-promoting transcription factors that may elevate metastatic potential.

Additional conserved regulators are relevant to breast cancer, although direct modulation of Jak/STAT activity has not been defined in all cases. The transcriptional repressor, BCL6, appears to be important to promote mesenchymal properties of breast cancer cells [158]. E-cadherin is often downregulated during cell invasion to allow detachment from the tumor, and BCL6 serves a transcriptional repressor of *E-cadherin* in breast cancer cells. In breast metastatic lesions, reduction of activated nuclear STAT5a levels correlated to increased BCL-6 cellular expression [159]. PTP1B is also involved in breast cancers, acting as an antiproliferative agent. PTP1B acts a negative regulator of both STAT5 and Jak2 activation in invasive breast cancer cell lines [160]. Breast cancer patients with distant metastases showed high levels of PTP1B protein [161]. Lastly, PIAS has not yet been shown to be a direct Jak/STAT signaling regulator in human breast cancer metastasis, but it is involved in the disease. Inhibition of the ligase activity of PIAS1 increased metastases to bone in mice after injection of human breast cancer cells [162]. Further investigation of these regulators of Jak/STAT signaling and of the pathway itself may shed more light on the intricate molecular mechanisms of breast cancer metastasis.

### 5.2. Jak/STAT Signaling in Prostate Cancer Metastasis

As with other carcinomas, Jak/STAT core components, IL-6, Jak2, and STAT5, and other key regulators of this pathway are implicated in promotion of prostate tumor growth and metastasis, and there is no highly effective treatment to cure metastatic disease. Activated STAT5a/b indicates a poor prognosis [163]. Abnormal STAT5 activation was detected in over 60% of distant prostate cancer metastasis, including over 80% of those in lymph nodes in human clinical samples [164]. Moreover, two human prostate cancer cell lines with metastatic potential (DU145 and PC-3) displayed a three-fold increase in cell migration in wound healing assays upon STAT5 activation. When DU145 cells expressing activated STAT5 were injected into mice, it resulted in eleven times more lung metastases compared to control injections without activated STAT. This indicates that STAT activation can drive metastasis. However, this mechanism might be dependent on Src kinases instead of Jak. STAT5 has been shown to interact with the androgen receptor, via its DNA binding domain, and protect it against proteosomal degradation, which can induce tumor growth in prostate cancer cells and may not require Jak [163]. The IL-6 receptor has also been shown to promote prostate cancer metastasis. Soluble IL-6 binds to gp130, which activates it, but the inhibition of this factor reduced cell migration of DU145 cells in scratch assays and, conversely, the increased soluble IL-6R expression in DU145 cells reduced their adhesion by 25% [165]. IL-6 also downregulated the Maspin tumor suppressor in prostate cancer cell lines.

Further evidence for activated Jak2-STAT5a/b signaling leading to metastasis comes from the findings that this pathway regulates EMT markers [166]. The activated signaling induced mesenchymal markers, including the transcription factor, Twist, and the stem cell factor, BMI1, a polycomb group repressor component, and repressed epithelial markers, including E-cadherin in human prostate cell lines, xenograft mouse models, and patient derived explant cultures. Interestingly, reduction of Twist suppressed the activated STAT pro-migration effects. STAT5 activation also prompted significant reduction of E-cadherin expression in xenographs using a cell line that normally has high levels of this adhesion molecule. The in vivo inoculation of DU145 prostate cancer cells with Jak2-STAT5a/b, activated through the expression of prolactin, increased tumor metastasis by 69% in mice. Additionally, inhibition of Jak2 with the drug AZD1480 blocked Jak2-STAT5a/b signaling and suppressed prostate tumor growth in both cell culture and mouse models [167]. Additional human Jak and STAT homologs, with partial similarity to Hop and STAT92E, also have implications in prostate cancer [168], which are not discussed here. Thus, clearly the IL6-Jak2-STAT5 axis plays a regulatory role in prostate cancer and metastasis.

Disruptions of key, conserved regulators of Jak/STAT signaling are also implicated in human prostate cancer. The SOCS family appears to be involved. SOCS1 caused significant reductions in wound closure and invasive behavior when it is stably expressed in prostate cancer cell lines [169]. In mouse models having metastatic tumors, none of the SOCS1-expressing mice had macro metastasis, unlike the controls, suggesting a role for SOCS1 in metastasis suppression. SOCS1 expression was significantly reduced in patients with metastatic prostate cancer, likely due to overexpression of a regulatory microRNA. SOCS1 likely acts on the MET and hepatocyte growth factor receptor tyrosine kinase, but may have other roles. These data make SOCS1 a strong candidate to be investigated as a Jak/STAT regulator in this context. GP130, a receptor subunit of IL-6, increased the invasiveness of prostate cancer cells and reduced E-cadherin levels in vitro [170]. Patients with aggressive prostate cancer had elevated levels of soluble GP130. Thus, GP130 might exert upregulation of Jak/STAT signaling to contribute to prostate cancer metastasis and proliferation.

Other typical Jak/STAT regulators may function differently than expected in prostate cancer. Although PTP1B inhibits STAT activity, other PTPs seem to promote tumorigenesis or metastasis. PTP1B is overexpressed at the protein level in clinical samples of prostate tumors [171]. The copy number of the gene encoding PTP1B was also increased by more than 20% in metastatic tumor samples. PTP1B silencing did not highly affect cell proliferation; however, it drastically impaired the migration and invasive properties of tumor cells in culture. These results suggest PTP1B functions independently from conventional Jak/STAT inhibition in prostate cancer metastasis. PIAS1 is another protein expected to reduce Jak/STAT activity. Elevated PIAS1 protein expression has been observed in primary tumors and metastatic lesions from prostate cancer patients [172]. PIAS1 expression increased further after chemotherapy in resistant cells. Short term inhibition of PIAS1 resulted in reduced cell proliferation, while long-term inhibition triggered apoptosis in vitro. Thus, like PTP1B, PIAS1 seems to have roles independent from suppressing proliferative Jak/STAT signaling. Although confirmation is needed to determine these mechanisms, it is interesting that multiple Jak/STAT signaling regulators are implicated in prostate cancer metastasis, even if they work through different modes of action.

### 5.3. Jak/STAT Promotes Border Cell Migration in the Ovary

The first evidence for Jak/STAT signaling promoting cell motility came from *Drosophila* border cells, a subset of somatic follicular cells in the adult ovary [23,24,60]. Egg chambers in the ovary, which will each give rise to one egg, develop as a set of germline cells surrounded by a somatic monolayer epithelium of follicle cells. (For general fly oogenesis patterning reviews, see [173,174]). At each pole of the developing egg chamber, polar cells form, which secrete Upd 1 and Upd 3 [4,19,175]. Early STAT signaling is critical in this population for regulating apoptosis and permitting the survival of two polar cells [176]. Secreted Upd acts as a morphogen to pattern the follicular epithelium [177,178]. In response to Upd, about 12 nearby cells activate STAT signaling, and approximately six maintain high levels of activation and become the motile border cells [23,24,175,178]. At a key time in egg development, these cells become motile, surround the non-motile polar cells, invade into the adjacent germline tissue, then migrate as a group to the oocyte, where they are required for patterning and egg structure. This chemotactic migration integrates signals from multiple signaling pathways, including EGF and PDGF/VEGF [179]. In addition to Jak/STAT’s requirement in the acquisition of border cell motility, the cell cluster’s continued migration relies on STAT activity [61]. A key effector of this migratory fate decision is the transcription factor Slow border cells (Slbo), a homolog of human CCAAT Enhancer Binding Protein Delta (CEBPD). Interestingly, not only is STAT activation necessary for cell migration, but it is sufficient among epithelial cells in the egg chamber to induce motility [24,60]. Ectopic or continuous overactivation of the pathway results in too many cells becoming migratory. Thus, the ovary provides an ideal context for identification of Jak/STAT pathway regulators of cell motility, and the border cells are an outstanding model system for investigating genes that promote migration, which include many that are activated in metastasis [180,181,182].

Border cell motility has been likened to an EMT event; however, border cells move as a collective that retain some epithelial character. In particular, apical–basal polarity components are required [183,184,185]. Additionally, while downregulation of E-cadherin is known as a classic marker of EMT, in border cells it must be maintained. E-cadherin is an important downstream target activated by STAT and Slbo in border cells. E-cadherin is highly concentrated, subcellularly, toward the center of the cluster, and it is required there to maintain cluster integrity [186,187], and probably to maintain association between the Upd-secreting polar cells and the motile border cells to maintain STAT pathway signaling. At the outer edges of the cells, E-cadherin is needed for traction to move over germline cells, but its protein level is lower, presumably due to cell adhesions being continuously remodeled. Some current studies suggest carcinomas may metastasize as mixed cell clusters similar to border cells [182,188,189,190,191].

Since high levels of Jak/STAT signaling results in additional motile border cells, loss of function mutations in negative regulators also yield this phenotype. Mutations in *Socs36E*, a Socs5 homolog, or protein tyrosine phosphatase, *Ptp61F*, a homolog of the human gene encoding PTP1B, both disrupt border cell migration by allowing too many cells to become motile, due to excessive Jak/STAT signaling [61,89,96]. Interestingly, *Socs36E* transcription is activated in response to STAT activity, functioning as an autoregulatory break on the signaling system [89]. Ptp61F is thought to reduce Jak/STAT signaling by dephosphorylation of the receptor or Jak, or both, which is also the case for the mammalian PTPs [71,96].

A number of additional Jak/STAT regulators involved in border cell migration have been identified through unbiased genetic screens and further characterized. In particular, numerous transcriptional regulators influence pathway output. The Bcl6 homolog Ken functions to promote sufficiently high Jak/STAT activity levels by transcriptionally suppressing a *Stat92E*-targeted microRNA, *mir-279* [192]. While a homologous microRNA has not been identified in humans, other microRNA regulators exist that target signaling components [135]. An additional transcription factor in this regulatory network in border cells is the Jak/STAT feedback inhibitor, Apontic, but the closest human homolog for this protein, the fibrinogen silencer binding protein, is not very well studied [178]. Apontic is activated by the Eyes absent (EYA) transcriptional factor, and promotes the expression of *mir-279* and *Socs36E* to keep Jak/STAT signaling from getting too high [89,193]. Recent studies have shown that a component of a chromatin remodeling complex, encoded by *Brahma*, genetically interacts with *Stat92E*, indicating that epigenetic regulation plays a key role in modulating the transcriptional output of the pathway in motile cells [96], as is the case in *Drosophila* testes stem cells [142,143,145,194].

In border cells, Jak/STAT regulation is also controlled by cellular trafficking mechanisms. Exocytic regulation of Upd release from polar cells requires Snap Receptor (SNARE) components, N-ethylmalemide-Sensitive Factor (NSF) and alpha-Soluble NSF attachment protein (αSNAP), which reset the SNARE complexes to permit vesicle trafficking [195]. Endocytic control regulates turnover of activated receptors, which can downregulate signaling [61,98,99]. Additional regulatory components for Jak/STAT signaling at the molecular and cell biological level are currently under investigation.

## 6. *Drosophila*-Jak/STAT Regulators Implicated in Human Metastatic Diseases

The power of *Drosophila* genetics lies in the ability to identify new components of a process in an unbiased way. This strategy has been fruitful in determining many Jak/STAT signaling regulators in the cell types discussed above, as well as in other contexts that are beyond the scope of this review. As is clear, Jak/STAT signaling is functionally and mechanistically conserved between flies and humans, and many regulators are shared between these pathways. However, more regulatory players have been characterized to act on *Drosophila* Jak/STAT signaling but are not yet connected to this pathway in humans. In the following section, we discuss the human homologs of positive and negative *Drosophila* Jak/STAT regulators in cancer progression and metastasis (see Table 1). In many cases, the mechanisms by which these regulators result in cancer are unknown. We propose that their known interactions with Jak/STAT signaling in *Drosophila* lays the groundwork to explore similar potential roles in different types of cells and to understand their involvement in cancers better.

### 6.1. Positive Drosophila Jak/STAT Regulators in Metastasis

Beside core components, multiple positive regulators of the *Drosophila* Jak/STAT signaling pathway have human homologs that are implicated in cancer progression, invasiveness, and/or metastasis (Table 1). The ovarian protein OCIAD1 is overexpressed in metastatic cancer tissue compared to primary ovarian tumors [196]. Overexpression of OCIAD1 in the presence of lysophosphatidic acid induced cell adhesion to collagen and laminin in human ovarian cancer cell lines. Thus, OCIAD1 is a regulator of ovarian cancer cell migration and metastasis, and its *Drosophila* homolog, Asrij, is a positive regulator of Jak/STAT signaling in hematopoiesis [197]. OCIAD2 is often associated with OCIAD1, and it also can promote STAT3 activation and cell migration in human cell culture [198]. This supports the hypothesis that OCIAD1 may upregulate metastatic potential via Jak/STAT regulation in human ovarian cancer.

Elevated levels of the Pleckstrin homology domain-interacting protein (PHIP) is predictive of distant metastasis and reduced survival in human melanoma [199]. Silencing PHIP in an ovarian cancer cell line reduced invasion into Matrigel by more than one-third, and reduced metastatic potential by about half in a mouse model. Given that the fly homolog of PHIP, BRWD3, is a known positive regulator of Jak/STAT in *Drosophila* [73], Jak/STAT involvement in PHIP-dependent metastasis is worth further investigation.

IGF2BP1 is an mRNA binding factor implicated in multiple cancers [200]. Silencing of the IGF2BP1 gene reduced cell proliferation, migration and invasiveness of a cervical cancer cell line and HeLa cells in wound healing and trans-well migration assays [201]. *MicroRNA 140-5p* targets IGF2BP1 in this tissue. In vitro assays showed that IGF2BP1 downregulation by another microRNA (*miR-150*) reduced migration and invasion of an osteosarcoma cell line [202]. Moreover, *miR-150* suppressed tumor growth in an osteosarcoma xenograft mouse model via repression of IGF2BP1. In both hepatocellular carcinoma and glioblastoma cell lines, downregulating IGF2BP1 via different microRNAs reduces tumor proliferation and invasion potential [203,204]. In patients, high expression of this protein was associated with lung cancer progression [205]. Thus, IGF2BP1 behaves as a pro-metastatic agent, consistent with a predicted role in promoting Jak/STAT activity, like its homolog (Imp) acts in fly testes [206].

PRDX4 is linked to cancer progression in multiple cell types. It is overexpressed in non-small cell lung cancer and prostate cancer [207,208]. Molecularly, it appears to be a thioredoxin peroxidase. The PRDX4 gene and protein are also upregulated in human glioblastoma multiforme (GBM) and mouse models of this disease [209]. In vitro suppression of PRDX4 caused the reduction of GBM stem cell-like proliferation and the prolonged survival in orthotopic transplantation to mouse. The PRDX4 homolog in flies, Jafrac2, promotes hemocyte overgrowth by upregulating Jak/STAT activity [210]. Further investigation is required to determine if PRDX4 has a role in metastasis through Jak/STAT signaling.

The transcription factor CEBPD acts as a pro-metastatic factor in several cancers. CEBPD overexpression increased urothelial cell migration and showed an increase in invasion potential in vitro [211]. Inhibition of MMP2 significantly blocked CEBPD-induced migration and invasion properties. Additionally, lung tumor metastasis was significantly lower when tumor cells were injected into a CEBPD-null mouse compared to a control. However, this suggests an indirect effect of CEBPD in metastasis. Overexpression of CEBPD in lymphatic cells increased cell migration in vitro, and conversely its repression inhibited cell motility. In flies, the CEBPD homolog, Slbo, is a key transcriptional target of activated STAT in border cells [24]; thus, we propose CEBPD may be activated and function analogously to promote migration during tumor metastasis.

### 6.2. Negative Drosophila Jak/STAT Regulators in Metastasis

As is clear, abnormal activation of Jak/STAT can result in tumorigenesis and metastasis. Thus, negative regulators of the pathway could have roles in disease progression as well, especially if they are lost or blocked. As many negative regulators have been characterized in *Drosophila*, these are worthy of attention. The human proteins BPTF, UBAP2, REST, and EYA2 have *Drosophila* counterparts that act to repress Jak/STAT signaling in various cell types (Table 1). Though changes in each of these factors are associated with human cancer progression and metastasis, a direct connection to Jak/STAT signaling is less clear.

The nucleosome-remodeling factor BPTF has been shown to be present in various cancers with different metastatic potentials. Examination of patient tumor samples indicated that BPTF is a suppressor of lung cancer metastasis to the brain [212]. In a cultured melanoma cell line, BPTF suppressed proliferative capacity and significantly reduced metastases upon intravenous injection in nude mice [213]. However, in a different melanoma line, increased BPTF expression induced cell proliferation [214]. Consistent with the latter, knockdown of BPTF in lung adenocarcinoma cell lines inhibited cell proliferation and lung cancer growth in mouse models [215]. In hepatocellular carcinoma patients, BPTF was associated with low E-cadherin levels, high tumor numbers, and more vascular invasion [216]. Colorectal cancer patients who had higher BPTF expression tended to have poor survival [217], which suggests BPTF promotes cancer progression. Given the repressive function of its *Drosophila* homolog (E(Bx)) in Jak/STAT signaling, we hypothesize that BPTF could be involved in human cancer metastasis, in part, by suppressing Jak/STAT signaling. However, BPTF can be linked with pro- or anti-metastatic roles, which may mean changes in its expression are indirect, that it has cell type specific effects, or that it may act in human cancer pathogenesis via multiple mechanisms.

UBAP2 contains a ubiquitin-associated domain, so is presumed to function in protein turnover. *UBAP2* was found to be overexpressed at the gene level in samples collected from castration-resistant prostate cancer patients [218]. Additionally, UBAP2 expression was significantly higher in advanced prostate cancer and was even higher in metastatic prostate cancer. In prostate cancer cell lines, reduction of *UBAP2* copy number significantly reduced cell growth. On the other hand, UBAP2 was lowly expressed in hepatocellular carcinoma (HCC) patient samples [219]. In HCC cell lines, knockdown of UBAP2 enhanced invasion, proliferation, and tumor growth in vivo. The *Drosophila* homolog Lig mutates to embryonic lethality, but has mainly been shown to affect Jak/STAT signaling in eye progenitor cell growth [220], suggesting that it participates in cell type-specific regulation. Thus, it seems that UBAP2 might have tissue dependent roles in cancer as well. Whether it acts via regulating Jak/STAT signaling in metastasis is worth further study.

Reduction of another putative Jak/STAT regulator, the transcription factor REST, increased LIN28A expression and tumor growth both in cell culture and in models in vivo [221]. The tumor samples that showed low REST expression also showed local invasion. REST is also a proposed clinical marker for advanced prostate cancer [222]. However, in spite of data suggesting that REST has an anti-proliferative role, there has not yet been demonstration of Jak/STAT signaling involvement in this function. The fly homolog of REST is Pzg, which functions both in steroid hormone signaling and Jak/STAT signaling, in part through modulating nucleosome remodeling [223]. Thus, REST’s function in cancer cells may also link to chromatin regulation. 

Lastly, the transcription factor EYA2 was shown to be misregulated in a number of cancers. In lung adenocarcinoma patient samples, it was upregulated, and upon its knockdown in cell culture, tumor growth and invasion potential were suppressed [224]. EGFR has been shown to activate EYA2 leading to breast cancer growth, EMT, invasion, and lung metastasis in vitro and in vivo [225]. In contrast, a majority of pancreatic adenocarcinoma patients showed loss of EYA2 in tumor cells [226]. Reduction of EYA2 in pancreatic cancer cell lines increased cell proliferation, and stable EYA2 expression reduced metastasis in mouse xenographs. In *Drosophila*, the corresponding protein Eya activates Apontic, which negatively regulates Jak/STAT signaling in ovarian follicle cells [193], but Eya has multiple other roles in different cell types. Further investigation on EYA2’s mode of action and the possible involvement of Jak/STAT signaling in cancer metastasis are interesting areas yet to be understood.

In summary, many of the human homologs of *Drosophila* positive Jak/STAT regulators are shown to be linked to human cancer progression and/or metastasis. Understanding their mode of action in cancer progression via, possibly, Jak/STAT signaling could provide us a new direction towards therapeutic interventions.

## 7. Outlook

The portrait of Jak/STAT signaling in human cancer metastasis is far from complete, and is obviously very complex. The description we provide here fortifies the idea that further explanation of Jak/STAT regulation in *Drosophila* is warranted and useful for uncovering new genes with roles in human disease. Interestingly, new levels of pathway regulation are becoming apparent in multiple *Drosophila* cell types. These findings suggest that future studies are needed to evaluate further vesicle trafficking regulation and epigenetic control of Jak/STAT activity and target gene expression. Additional drug screening in *Drosophila* is also likely to be very informative. Further studies of the candidates described here and new ones will likely shed light on the regulation of cancer metastasis by Jak/STAT signaling or additional linked pathways.

## Figures and Tables

**Table 1:** Roles for *Drosophila* Jak/STAT signaling components and their human homologs in development and cancer metastasis.

*Drosophila* Jak/STAT Component	*Drosophila* Cell Type/Tissue	Human Homolog	Metastatic Cancer Type
Signal Transducer and Activator of Transcription92E (STAT92E)	Brain [227] Embryo [3,7] Eyes [51,53] Hindgut [228,229] Lymph Glands [117] Ovaries [24,60,176,177,230] Primordial Germ Cells [231,232,233] Testes [58,59] Trachea [229] Wing Disc [52]	Signal Transducer and Activator of Transcription 5b (STAT5b)	Brain Cancer [234] Breast Cancer [149,150] Colorectal Cancer [235,236,237] Melanoma [238] Pancreatic Cancer [239] Prostate Cancer [163,164]
Hopscotch (Hop)	Brain [227] Embryo [7,28] Eyes [53,240] Haltere Disc [52] Hemocytes [10] Hindgut [229] Leg Disc [52] Lymph Glands [116] Somatic Muscle [241] Ovaries [23,24,60,177,242] Trachea [229] Testes [58] Wing Disc [52]	Janus Kinase 2 (Jak2)	Breast Cancer [151,153,157] Bone Cancer [243] Cervical Cancer [244] Colorectal Cancer [245] Melanoma [246] Pancreatic Cancer [247] Prostate Cancer [166,167]
Domeless (Dome)	Brain [227] Embryo [5,6] Eye [248] Hindgut [228] Lymph Gland [120,126] Ovaries [23,177,249] Trachea [6] Wing [52]	Interleukin 6 Receptor (IL-6R)	Breast Cancer [154] Bone Cancer [250] Colorectal Cancer [251,252,253] Hepatocellular Carcinoma [254] Ovarian Cancer [255] Pancreatic Cancer [256] Prostate Cancer [165]
Eye Transformer (Et)	Eye [26] Intestines [26] Lymph Gland [27]	Glycoprotein 130 (GP130)	Melanoma [257] Prostate Cancer [170]
Ken and Barbie (Ken)	Embryo [258] Eyes [259] Genitalia [258,259] Ovaries [192] Testes [141]	B Cell CLL/Lymphoma 6 (BCL6)	Breast Cancer [159]
Protein Tyrosine Phosphatase 61F (Ptp61F)	Eyes [78] Ovaries [96] Testes [141]	Protein Tyrosine Phosphatase 1B (PTP1B)	Breast Cancer [160,161] Colorectal Cancer [260] Esophageal Cancer [261] Lung Cancer [262] Melanoma [263] Ovarian Cancer [264] Prostate Cancer [171]
Protein Inhibitor of Activated STAT (Pias) (Su(var) 2-10)	Eyes [78] Hemocytes [78] Ovaries [23]	Protein Inhibitor of Activated STAT 1 (PIAS1)	Breast Cancer [162] Gastric Cancer [265] Prostate Cancer [172]
Suppressor of Cytokine Signaling 44A (Socs44A)	Wings [82]	Suppressor of Cytokine Signaling 1 (SOCS1)	Breast Cancer [156] Colorectal Cancer [266] Hepatocellular Carcinoma [267] Melanoma [268] Prostate Cancer [169]
Suppressor of Cytokine Signaling 36E (Socs36E)	Notum [83] Ovaries [61,89] Testes [85,138,139] Wing Disc [82,83,84,87]	Suppressor of Cytokine Signaling 5 (SOCS5)	Colorectal Cancer [269] Liver Cancer [270]
Enhancer of Bithorax (E(Bx))	Testes [271]	Bromodomain PHD Finger Transcription Factor (BPTF)	Brain Cancer [212] Colorectal Cancer [217] Hepatocellular Carcinoma [216] Lung Cancer [215] Melanoma [213,214]
Lingerer (Lig)	Eyes [220] Imaginal Discs [220] Ovaries [272]	Ubiquitin Associated Protein 2 (UBAP2)	Hepatocellular Carcinoma [219] Prostate Cancer [218]
Putzig (Pzg)	Heart [273] Wing Disc [223]	RE1 Silencing Transcription Factor (REST)	Breast Cancer [221] Prostate Cancer [222]
Eyes Absent (Eya)	Eyes [274,275,276] Salivary Gland [277] Somatic Muscle [241] Ovaries [193,249,278] Testes [279]	Eyes Absent 2 (EYA2)	Brain Cancer [280] Breast Cancer [281,282]
Asrij (Arj)	Head [283] Hemocyte [119,120] Lymph Gland [197] Trachea [119]	Ovarian Cancer Immunoreactive Antigen Domain Containing 1 (OCIAD1)	Ovarian Cancer [196]
Bromodomain and WD Repeat Containing Protein 3 (BRWD3)	Eyes [284] Heart [273] Midgut [285] Salivary Gland [285] Testes [286]	Pleckstrin Homology Domain Interacting Protein (PHIP)	Melanoma [199,287]
IGF-II-mRNA-Binding Protein (Imp)	Head [283] Ovaries [288] Testes [206,279]	Insulin-like Growth Factor II Binding Protein 1 (IGF2BP1)	Bone Cancer [202] Cervical Cancer [201] Hepatocellular Carcinoma [289,290,291] Lung Cancer [205]
Thioredoxin Peroxidase 2 (Jafrac2)	Head [283] Heart [273] Hemolymph [210]	Peroxiredoxin 4 (PRDX4)	Brain Cancer [209] Lung Cancer [207]
Slow Border Cells (Slbo)	Ovaries [292]	CCAAT Enhancer Binding Protein Delta (CEBPD)	Lung Cancer [293] Nasopharyngeal Carcinoma [294] Urothelial Cancer [211]
C-Terminal Src Kinase (Csk)	Eyes [295,296] Imaginal Discs [297] Ovaries [298]	C-Terminal Src Kinase (CSK)	Colon Cancer [299,300]

The left side of the table shows canonical *Drosophila* Jak/STAT components and tissues in which they are required or highly expressed. The right side of the table lists the closest human homologs and the types of metastatic tumors in which they are involved.

## References

[1] Stark G.R., Darnell J.E. (2012). The JAK-STAT pathway at twenty. Immunity.

[2] O’Shea J.J., Murray P.J. (2008). Cytokine signaling modules in inflammatory responses. Immunity.

[3] Yan R., Small S., Desplan C., Dearolf C.R., Darnell J.E. (1996). Identification of a Stat gene that functions in *Drosophila* development. Cell.

[4] Harrison D.A., McCoon P.E., Binari R., Gilman M., Perrimon N. (1998). *Drosophila* unpaired encodes a secreted protein that activates the JAK signaling pathway. Genes Dev..

[5] Chen H.W., Chen X., Oh S.W., Marinissen M.J., Gutkind J.S., Hou S.X. (2002). mom identifies a receptor for the *Drosophila* JAK/STAT signal transduction pathway and encodes a protein distantly related to the mammalian cytokine receptor family. Genes Dev..

[6] Brown S., Hu N., Hombria J.C. (2001). Identification of the first invertebrate interleukin JAK/STAT receptor, the *Drosophila* gene domeless. Curr. Biol..

[7] Hou X.S., Melnick M.B., Perrimon N. (1996). Marelle acts downstream of the *Drosophila* HOP/JAK kinase and encodes a protein similar to the mammalian STATs. Cell.

[8] Luo H., Hanratty W.P., Dearolf C.R. (1995). An amino acid substitution in the *Drosophila* hopTum-l Jak kinase causes leukemia-like hematopoietic defects. EMBO J..

[9] Luo H., Rose P., Barber D., Hanratty W.P., Lee S., Roberts T.M., D’Andrea A.D., Dearolf C.R. (1997). Mutation in the Jak kinase JH2 domain hyperactivates *Drosophila* and mammalian Jak-Stat pathways. Mol. Cell. Biol..

[10] Hanratty W.P., Dearolf C.R. (1993). The *Drosophila* Tumorous-lethal hematopoietic oncogene is a dominant mutation in the hopscotch locus. Mol. Gen. Genet..

[11] Harrison D.A., Binari R., Nahreini T.S., Gilman M., Perrimon N. (1995). Activation of a *Drosophila* Janus kinase (JAK) causes hematopoietic neoplasia and developmental defects. EMBO J..

[12] Lacronique V., Boureux A., Valle V.D., Poirel H., Quang C.T., Mauchauffe M., Berthou C., Lessard M., Berger R., Ghysdael J. (1997). A TEL-JAK2 fusion protein with constitutive kinase activity in human leukemia. Science.

[13] Peeters P., Raynaud S.D., Cools J., Wlodarska I., Grosgeorge J., Philip P., Monpoux F., Van Rompaey L., Baens M., Van den Berghe H. (1997). Fusion of TEL, the ETS-variant gene 6 (ETV6), to the receptor-associated kinase JAK2 as a result of t(9;12) in a lymphoid and t(9;15;12) in a myeloid leukemia. Blood.

[14] Ward A.C., Touw I., Yoshimura A. (2000). The Jak-Stat pathway in normal and perturbed hematopoiesis. Blood.

[15] Bromberg J., Darnell J.E. (2000). The role of STATs in transcriptional control and their impact on cellular function. Oncogene.

[16] Schindler C., Plumlee C. (2008). Inteferons pen the JAK-STAT pathway. Semin. Cell Dev. Biol..

[17] Arbouzova N.I., Zeidler M.P. (2006). JAK/STAT signalling in *Drosophila*: Insights into conserved regulatory and cellular functions. Development.

[18] Hombria J.C., Brown S., Hader S., Zeidler M.P. (2005). Characterisation of Upd2, a *Drosophila* JAK/STAT pathway ligand. Dev. Biol..

[19] Wang L., Sexton T.R., Venard C., Giedt M., Guo Q., Chen Q., Harrison D.A. (2014). Pleiotropy of the *Drosophila* JAK pathway cytokine Unpaired 3 in development and aging. Dev. Biol..

[20] Wright V.M., Vogt K.L., Smythe E., Zeidler M.P. (2011). Differential activities of the *Drosophila* JAK/STAT pathway ligands Upd, Upd2 and Upd3. Cell Signal..

[21] Levy D.E., Darnell J.E. (2002). Stats: Transcriptional control and biological impact. Nat. Rev. Mol. Cell Biol..

[22] Murray P.J. (2007). The JAK-STAT signaling pathway: Input and output integration. J. Immunol..

[23] Ghiglione C., Devergne O., Georgenthum E., Carballes F., Medioni C., Cerezo D., Noselli S. (2002). The *Drosophila* cytokine receptor Domeless controls border cell migration and epithelial polarization during oogenesis. Development.

[24] Silver D.L., Montell D.J. (2001). Paracrine signaling through the JAK/STAT pathway activates invasive behavior of ovarian epithelial cells in *Drosophila*. Cell.

[25] Fisher K.H., Stec W., Brown S., Zeidler M.P. (2016). Mechanisms of JAK/STAT pathway negative regulation by the short coreceptor Eye Transformer/Latran. Mol. Biol. Cell.

[26] Kallio J., Myllymaki H., Gronholm J., Armstrong M., Vanha-aho L.M., Makinen L., Silvennoinen O., Valanne S., Ramet M. (2010). Eye transformer is a negative regulator of *Drosophila* JAK/STAT signaling. FASEB J..

[27] Makki R., Meister M., Pennetier D., Ubeda J.M., Braun A., Daburon V., Krzemien J., Bourbon H.M., Zhou R., Vincent A. (2010). A short receptor downregulates JAK/STAT signalling to control the *Drosophila* cellular immune response. PLoS Biol..

[28] Perrimon N., Mahowald A.P. (1986). l(1)hopscotch, A larval-pupal zygotic lethal with a specific maternal effect on segmentation in *Drosophila*. Dev. Biol..

[29] Yamaoka K., Saharinen P., Pesu M., Holt V.E., Silvennoinen O., O’Shea J.J. (2004). The Janus kinases (Jaks). Genome Biol..

[30] Sweitzer S.M., Calvo S., Kraus M.H., Finbloom D.S., Larner A.C. (1995). Characterization of a Stat-like DNA binding activity in *Drosophila* melanogaster. J. Biol. Chem..

[31] Silver-Morse L., Li W.X. (2013). JAK-STAT in heterochromatin and genome stability. JAKSTAT.

[32] Brown S., Zeidler M.P. (2008). Unphosphorylated STATs go nuclear. Curr. Opin. Genet. Dev..

[33] Shi S., Calhoun H.C., Xia F., Li J., Le L., Li W.X. (2006). JAK signaling globally counteracts heterochromatic gene silencing. Nat. Genet..

[34] Yang J., Stark G.R. (2008). Roles of unphosphorylated STATs in signaling. Cell Res..

[35] Wingelhofer B., Neubauer H.A., Valent P., Han X., Constantinescu S.N., Gunning P.T., Muller M., Moriggl R. (2018). Implications of STAT3 and STAT5 signaling on gene regulation and chromatin remodeling in hematopoietic cancer. Leukemia.

[36] Casanova J.L., Holland S.M., Notarangelo L.D. (2012). Inborn errors of human JAKs and STATs. Immunity.

[37] Valentino L., Pierre J. (2006). JAK/STAT signal transduction: Regulators and implication in hematological malignancies. Biochem. Pharmacol..

[38] Boudny V., Kovarik J. (2002). JAK/STAT signaling pathways and cancer. Janus kinases/signal transducers and activators of transcription. Neoplasma.

[39] Dorritie K.A., Redner R.L., Johnson D.E. (2014). STAT transcription factors in normal and cancer stem cells. Adv. Biol. Regul..

[40] Akira S. (1999). Functional roles of STAT family proteins: Lessons from knockout mice. Stem Cells.

[41] Campbell I.L. (2005). Cytokine-mediated inflammation, tumorigenesis, and disease-associated JAK/STAT/SOCS signaling circuits in the CNS. Brain Res. Brain Res. Rev..

[42] Hennighausen L., Robinson G.W. (2008). Interpretation of cytokine signaling through the transcription factors STAT5A and STAT5B. Genes Dev..

[43] Del Valle Rodriguez A., Didiano D., Desplan C. (2011). Power tools for gene expression and clonal analysis in *Drosophila*. Nat. Methods.

[44] Chen Q., Giedt M., Tang L., Harrison D.A. (2014). Tools and methods for studying the *Drosophila* JAK/STAT pathway. Methods.

[45] Jinks T.M., Polydorides A.D., Calhoun G., Schedl P. (2000). The JAK/STAT signaling pathway is required for the initial choice of sexual identity in *Drosophila* melanogaster. Mol. Cell.

[46] Sefton L., Timmer J.R., Zhang Y., Beranger F., Cline T.W. (2000). An extracellular activator of the *Drosophila* JAK/STAT pathway is a sex-determination signal element. Nature.

[47] Arbouzova N.I., Bach E.A., Zeidler M.P. (2006). Ken & barbie selectively regulates the expression of a subset of Jak/STAT pathway target genes. Curr. Biol..

[48] Wawersik M., Milutinovich A., Casper A.L., Matunis E., Williams B., Van Doren M. (2005). Somatic control of germline sexual development is mediated by the JAK/STAT pathway. Nature.

[49] Agaisse H., Perrimon N. (2004). The roles of JAK/STAT signaling in *Drosophila* immune responses. Immunol. Rev..

[50] Minakhina S., Steward R. (2006). Melanotic mutants in *Drosophila*: Pathways and phenotypes. Genetics.

[51] Ekas L.A., Baeg G.H., Flaherty M.S., Ayala-Camargo A., Bach E.A. (2006). JAK/STAT signaling promotes regional specification by negatively regulating wingless expression in *Drosophila*. Development.

[52] Recasens-Alvarez C., Ferreira A., Milan M. (2017). JAK/STAT controls organ size and fate specification by regulating morphogen production and signalling. Nat. Commun..

[53] Zeidler M.P., Perrimon N., Strutt D.I. (1999). Polarity determination in the *Drosophila* eye: A novel role for unpaired and JAK/STAT signaling. Genes Dev..

[54] Lengyel J.A., Iwaki D.D. (2002). It takes guts: The *Drosophila* hindgut as a model system for organogenesis. Dev. Biol..

[55] Nagy P., Kovacs L., Sandor G.O., Juhasz G. (2016). Stem-cell-specific endocytic degradation defects lead to intestinal dysplasia in *Drosophila*. Dis. Model. Mech..

[56] Buchon N., Broderick N.A., Poidevin M., Pradervand S., Lemaitre B. (2009). *Drosophila* intestinal response to bacterial infection: Activation of host defense and stem cell proliferation. Cell Host Microbe.

[57] Leatherman J.L., Dinardo S. (2010). Germline self-renewal requires cyst stem cells and stat regulates niche adhesion in *Drosophila* testes. Nat. Cell Biol..

[58] Tulina N., Matunis E. (2001). Control of stem cell self-renewal in *Drosophila* spermatogenesis by JAK-STAT signaling. Science.

[59] Kiger A.A., Jones D.L., Schulz C., Rogers M.B., Fuller M.T. (2001). Stem cell self-renewal specified by JAK-STAT activation in response to a support cell cue. Science.

[60] Beccari S., Teixeira L., Rorth P. (2002). The JAK/STAT pathway is required for border cell migration during *Drosophila* oogenesis. Mech. Dev..

[61] Silver D.L., Geisbrecht E.R., Montell D.J. (2005). Requirement for JAK/STAT signaling throughout border cell migration in *Drosophila*. Development.

[62] Amoyel M., Bach E.A. (2012). Functions of the *Drosophila* JAK-STAT pathway: Lessons from stem cells. JAKSTAT.

[63] Hombria J.C., Brown S. (2002). The fertile field of *Drosophila* Jak/STAT signalling. Curr. Biol..

[64] Fossett N. (2013). Signal transduction pathways, intrinsic regulators, and the control of cell fate choice. Biochim. Biophys. Acta.

[65] Bausek N. (2013). JAK-STAT signaling in stem cells and their niches in *Drosophila*. JAKSTAT.

[66] Amoyel M., Anderson A.M., Bach E.A. (2014). JAK/STAT pathway dysregulation in tumors: A *Drosophila* perspective. Semin. Cell Dev. Biol..

[67] Morin-Poulard I., Vincent A., Crozatier M. (2013). The *Drosophila* JAK-STAT pathway in blood cell formation and immunity. JAKSTAT.

[68] Hombria J.C., Sotillos S. (2013). JAK-STAT pathway in *Drosophila* morphogenesis: From organ selector to cell behavior regulator. JAKSTAT.

[69] Zoranovic T., Grmai L., Bach E.A. (2013). Regulation of proliferation, cell competition, and cellular growth by the *Drosophila* JAK-STAT pathway. JAKSTAT.

[70] Keebaugh E.S., Schlenke T.A. (2014). Insights from natural host-parasite interactions: The *Drosophila* model. Dev. Comp. Immunol..

[71] Baeg G.H., Zhou R., Perrimon N. (2005). Genome-wide RNAi analysis of JAK/STAT signaling components in *Drosophila*. Genes Dev..

[72] Fisher K.H., Wright V.M., Taylor A., Zeidler M.P., Brown S. (2012). Advances in genome-wide RNAi cellular screens: A case study using the *Drosophila* JAK/STAT pathway. BMC Genom..

[73] Muller P., Kuttenkeuler D., Gesellchen V., Zeidler M.P., Boutros M. (2005). Identification of JAK/STAT signalling components by genome-wide RNA interference. Nature.

[74] Rakesh K., Agrawal D.K. (2005). Controlling cytokine signaling by constitutive inhibitors. Biochem. Pharmacol..

[75] Starr R., Hilton D.J. (1999). Negative regulation of the JAK/STAT pathway. Bioessays.

[76] Wormald S., Hilton D.J. (2004). Inhibitors of cytokine signal transduction. J. Biol. Chem..

[77] Rytinki M.M., Kaikkonen S., Pehkonen P., Jaaskelainen T., Palvimo J.J. (2009). PIAS proteins: Pleiotropic interactors associated with SUMO. Cell. Mol. Life Sci..

[78] Betz A., Lampen N., Martinek S., Young M.W., Darnell J.E. (2001). A *Drosophila* PIAS homologue negatively regulates stat92E. Proc. Natl. Acad. Sci. USA.

[79] Zeidler M.P., Bausek N. (2013). The *Drosophila* JAK-STAT pathway. JAKSTAT.

[80] Alexander W.S., Hilton D.J. (2004). The role of suppressors of cytokine signaling (SOCS) proteins in regulation of the immune response. Annu. Rev. Immunol..

[81] Croker B.A., Kiu H., Nicholson S.E. (2008). SOCS regulation of the JAK/STAT signalling pathway. Semin. Cell Dev. Biol..

[82] Rawlings J.S., Rennebeck G., Harrison S.M., Xi R., Harrison D.A. (2004). Two *Drosophila* suppressors of cytokine signaling (SOCS) differentially regulate JAK and EGFR pathway activities. BMC Cell Biol..

[83] Callus B.A., Mathey-Prevot B. (2002). SOCS36E, a novel *Drosophila* SOCS protein, suppresses JAK/STAT and EGF-R signalling in the imaginal wing disc. Oncogene.

[84] Karsten P., Hader S., Zeidler M.P. (2002). Cloning and expression of *Drosophila* SOCS36E and its potential regulation by the JAK/STAT pathway. Mech. Dev..

[85] Amoyel M., Anderson J., Suisse A., Glasner J., Bach E.A. (2016). Socs36E Controls Niche Competition by Repressing MAPK Signaling in the *Drosophila* Testis. PLoS Genet..

[86] Almudi I., Stocker H., Hafen E., Corominas M., Serras F. (2009). SOCS36E specifically interferes with Sevenless signaling during *Drosophila* eye development. Dev. Biol..

[87] Herranz H., Hong X., Hung N.T., Voorhoeve P.M., Cohen S.M. (2012). Oncogenic cooperation between SOCS family proteins and EGFR identified using a *Drosophila* epithelial transformation model. Genes Dev..

[88] Monahan A.J., Starz-Gaiano M. (2015). Socs36E limits STAT signaling via Cullin2 and a SOCS-box independent mechanism in the *Drosophila* egg chamber. Mech. Dev..

[89] Monahan A.J., Starz-Gaiano M. (2013). Socs36E attenuates STAT signaling to optimize motile cell specification in the *Drosophila* ovary. Dev. Biol..

[90] Matsumoto A., Masuhara M., Mitsui K., Yokouchi M., Ohtsubo M., Misawa H., Miyajima A., Yoshimura A. (1997). CIS, a cytokine inducible SH2 protein, is a target of the JAK-STAT5 pathway and modulates STAT5 activation. Blood.

[91] Matsumoto A., Seki Y., Kubo M., Ohtsuka S., Suzuki A., Hayashi I., Tsuji K., Nakahata T., Okabe M., Yamada S. (1999). Suppression of STAT5 functions in liver, mammary glands, and T cells in cytokine-inducible SH2-containing protein 1 transgenic mice. Mol. Cell. Biol..

[92] Endo T.A., Masuhara M., Yokouchi M., Suzuki R., Sakamoto H., Mitsui K., Matsumoto A., Tanimura S., Ohtsubo M., Misawa H. (1997). A new protein containing an SH2 domain that inhibits JAK kinases. Nature.

[93] Aoki N., Matsuda T. (2000). A cytosolic protein-tyrosine phosphatase PTP1B specifically dephosphorylates and deactivates prolactin-activated STAT5a and STAT5b. J. Biol. Chem..

[94] Myers M.P., Andersen J.N., Cheng A., Tremblay M.L., Horvath C.M., Parisien J.P., Salmeen A., Barford D., Tonks N.K. (2001). TYK2 and JAK2 are substrates of protein-tyrosine phosphatase 1B. J. Biol. Chem..

[95] Mustelin T., Vang T., Bottini N. (2005). Protein tyrosine phosphatases and the immune response. Nat. Rev. Immunol..

[96] Saadin A., Starz-Gaiano M. (2016). Identification of Novel Regulators of the JAK/STAT Signaling Pathway that Control Border Cell Migration in the *Drosophila* Ovary. G3.

[97] Strous G.J., van Kerkhof P., Govers R., Ciechanover A., Schwartz A.L. (1996). The ubiquitin conjugation system is required for ligand-induced endocytosis and degradation of the growth hormone receptor. EMBO J..

[98] Vidal O.M., Stec W., Bausek N., Smythe E., Zeidler M.P. (2010). Negative regulation of *Drosophila* JAK-STAT signalling by endocytic trafficking. J. Cell Sci..

[99] Devergne O., Ghiglione C., Noselli S. (2007). The endocytic control of JAK/STAT signalling in *Drosophila*. J. Cell Sci..

[100] Kurgonaite K., Gandhi H., Kurth T., Pautot S., Schwille P., Weidemann T., Bokel C. (2015). Essential role of endocytosis for interleukin-4-receptor-mediated JAK/STAT signalling. J. Cell Sci..

[101] Ren W., Zhang Y., Li M., Wu L., Wang G., Baeg G.H., You J., Li Z., Lin X. (2015). Windpipe controls *Drosophila* intestinal homeostasis by regulating JAK/STAT pathway via promoting receptor endocytosis and lysosomal degradation. PLoS Genet..

[102] Radtke S., Wuller S., Yang X.P., Lippok B.E., Mutze B., Mais C., de Leur H.S., Bode J.G., Gaestel M., Heinrich P.C. (2010). Cross-regulation of cytokine signalling: Pro-inflammatory cytokines restrict IL-6 signalling through receptor internalisation and degradation. J. Cell Sci..

[103] Schepers H., Wierenga A.T., Vellenga E., Schuringa J.J. (2012). STAT5-mediated self-renewal of normal hematopoietic and leukemic stem cells. JAKSTAT.

[104] Kato Y., Iwama A., Tadokoro Y., Shimoda K., Minoguchi M., Akira S., Tanaka M., Miyajima A., Kitamura T., Nakauchi H. (2005). Selective activation of STAT5 unveils its role in stem cell self-renewal in normal and leukemic hematopoiesis. J. Exp. Med..

[105] Staerk J., Kallin A., Royer Y., Diaconu C.C., Dusa A., Demoulin J.B., Vainchenker W., Constantinescu S.N. (2007). JAK2, the JAK2 V617F mutant and cytokine receptors. Pathol. Biol..

[106] Staerk J., Kallin A., Demoulin J.B., Vainchenker W., Constantinescu S.N. (2005). JAK1 and Tyk2 activation by the homologous polycythemia vera JAK2 V617F mutation: Cross-talk with IGF1 receptor. J. Biol. Chem..

[107] Faderl S., Ferrajoli A., Harris D., Van Q., Priebe W., Estrov Z. (2005). WP-1034, a novel JAK-STAT inhibitor, with proapoptotic and antileukemic activity in acute myeloid leukemia (AML). Anticancer Res..

[108] Meydan N., Grunberger T., Dadi H., Shahar M., Arpaia E., Lapidot Z., Leeder J.S., Freedman M., Cohen A., Gazit A. (1996). Inhibition of acute lymphoblastic leukaemia by a Jak-2 inhibitor. Nature.

[109] Ghoreschi K., Jesson M.I., Li X., Lee J.L., Ghosh S., Alsup J.W., Warner J.D., Tanaka M., Steward-Tharp S.M., Gadina M. (2011). Modulation of innate and adaptive immune responses by tofacitinib (CP-690,550). J. Immunol..

[110] Lanot R., Zachary D., Holder F., Meister M. (2001). Postembryonic hematopoiesis in *Drosophila*. Dev. Biol..

[111] Krzemien J., Dubois L., Makki R., Meister M., Vincent A., Crozatier M. (2007). Control of blood cell homeostasis in *Drosophila* larvae by the posterior signalling centre. Nature.

[112] Bazzi W., Cattenoz P.B., Delaporte C., Dasari V., Sakr R., Yuasa Y., Giangrande A. (2018). Embryonic hematopoiesis modulates the inflammatory response and larval hematopoiesis in *Drosophila*. eLife.

[113] Munier A.I., Doucet D., Perrodou E., Zachary D., Meister M., Hoffmann J.A., Janeway C.A., Lagueux M. (2002). PVF2, a PDGF/VEGF-like growth factor, induces hemocyte proliferation in *Drosophila* larvae. EMBO Rep..

[114] Bruckner K., Kockel L., Duchek P., Luque C.M., Rorth P., Perrimon N. (2004). The PDGF/VEGF receptor controls blood cell survival in *Drosophila*. Dev. Cell.

[115] Anderson A.M., Bailetti A.A., Rodkin E., De A., Bach E.A. (2017). A Genetic Screen Reveals an Unexpected Role for Yorkie Signaling in JAK/STAT-Dependent Hematopoietic Malignancies in *Drosophila* melanogaster. G3.

[116] Sorrentino R.P., Tokusumi T., Schulz R.A. (2007). The Friend of GATA protein U-shaped functions as a hematopoietic tumor suppressor in *Drosophila*. Dev. Biol..

[117] Gao H., Wu X., Fossett N. (2009). Upregulation of the *Drosophila* Friend of GATA gene U-shaped by JAK/STAT signaling maintains lymph gland prohemocyte potency. Mol. Cell. Biol..

[118] Minakhina S., Tan W., Steward R. (2011). JAK/STAT and the GATA factor Pannier control hemocyte maturation and differentiation in *Drosophila*. Dev. Biol..

[119] Inamdar M.S. (2003). *Drosophila* asrij is expressed in pole cells, trachea and hemocytes. Dev. Genes Evol..

[120] Khadilkar R.J., Rodrigues D., Mote R.D., Sinha A.R., Kulkarni V., Magadi S.S., Inamdar M.S. (2014). ARF1-GTP regulates Asrij to provide endocytic control of *Drosophila* blood cell homeostasis. Proc. Natl. Acad. Sci. USA.

[121] Bina S., Wright V.M., Fisher K.H., Milo M., Zeidler M.P. (2010). Transcriptional targets of *Drosophila* JAK/STAT pathway signalling as effectors of haematopoietic tumour formation. EMBO Rep..

[122] Ghosh S., Singh A., Mandal S., Mandal L. (2015). Active hematopoietic hubs in *Drosophila* adults generate hemocytes and contribute to immune response. Dev. Cell.

[123] Zeidler M.P., Bach E.A., Perrimon N. (2000). The roles of the *Drosophila* JAK/STAT pathway. Oncogene.

[124] Agaisse H., Petersen U.M., Boutros M., Mathey-Prevot B., Perrimon N. (2003). Signaling role of hemocytes in *Drosophila* JAK/STAT-dependent response to septic injury. Dev. Cell.

[125] Dearolf C.R. (1998). Fruit fly “leukemia”. Biochim. Biophys. Acta.

[126] Sinenko S.A., Hung T., Moroz T., Tran Q.M., Sidhu S., Cheney M.D., Speck N.A., Banerjee U. (2010). Genetic manipulation of AML1-ETO-induced expansion of hematopoietic precursors in a *Drosophila* model. Blood.

[127] Kim B.H., Oh S.R., Yin C.H., Lee S., Kim E.A., Kim M.S., Sandoval C., Jayabose S., Bach E.A., Lee H.K. (2010). MS-1020 is a novel small molecule that selectively inhibits JAK3 activity. Br. J. Haematol..

[128] Kim B.H., Yin C.H., Guo Q., Bach E.A., Lee H., Sandoval C., Jayabose S., Ulaczyk-Lesanko A., Hall D.G., Baeg G.H. (2008). A small-molecule compound identified through a cell-based screening inhibits JAK/STAT pathway signaling in human cancer cells. Mol. Cancer Ther..

[129] Thomas S., Fisher K., Snowden J., Danson S., Brown S., Zeidler M. (2015). Effect of methotrexate on JAK/STAT pathway activation in myeloproliferative neoplasms. Lancet.

[130] Thomas S., Fisher K.H., Snowden J.A., Danson S.J., Brown S., Zeidler M.P. (2015). Methotrexate Is a JAK/STAT Pathway Inhibitor. PLoS ONE.

[131] Eggert U.S., Kiger A.A., Richter C., Perlman Z.E., Perrimon N., Mitchison T.J., Field C.M. (2004). Parallel chemical genetic and genome-wide RNAi screens identify cytokinesis inhibitors and targets. PLoS Biol..

[132] Markstein M., Dettorre S., Cho J., Neumuller R.A., Craig-Muller S., Perrimon N. (2014). Systematic screen of chemotherapeutics in *Drosophila* stem cell tumors. Proc. Natl. Acad. Sci. USA.

[133] Levy D.E., Gilliland D.G. (2000). Divergent roles of STAT1 and STAT5 in malignancy as revealed by gene disruptions in mice. Oncogene.

[134] Calo V., Migliavacca M., Bazan V., Macaluso M., Buscemi M., Gebbia N., Russo A. (2003). STAT proteins: From normal control of cellular events to tumorigenesis. J. Cell. Physiol..

[135] Yu H., Lee H., Herrmann A., Buettner R., Jove R. (2014). Revisiting STAT3 signalling in cancer: New and unexpected biological functions. Nat. Rev. Cancer.

[136] Islam F., Gopalan V., Smith R.A., Lam A.K. (2015). Translational potential of cancer stem cells: A review of the detection of cancer stem cells and their roles in cancer recurrence and cancer treatment. Exp. Cell Res..

[137] Issigonis M., Matunis E. (2011). SnapShot: Stem cell niches of the *Drosophila* testis and ovary. Cell.

[138] Singh S.R., Zheng Z., Wang H., Oh S.W., Chen X., Hou S.X. (2010). Competitiveness for the niche and mutual dependence of the germline and somatic stem cells in the *Drosophila* testis are regulated by the JAK/STAT signaling. J. Cell. Physiol..

[139] Issigonis M., Tulina N., de Cuevas M., Brawley C., Sandler L., Matunis E. (2009). JAK-STAT signal inhibition regulates competition in the *Drosophila* testis stem cell niche. Science.

[140] Leatherman J.L., Dinardo S. (2008). Zfh-1 controls somatic stem cell self-renewal in the *Drosophila* testis and nonautonomously influences germline stem cell self-renewal. Cell Stem Cell.

[141] Issigonis M., Matunis E. (2012). The *Drosophila* BCL6 homolog Ken and Barbie promotes somatic stem cell self-renewal in the testis niche. Dev. Biol..

[142] Cherry C.M., Matunis E.L. (2010). Epigenetic regulation of stem cell maintenance in the *Drosophila* testis via the nucleosome-remodeling factor NURF. Cell Stem Cell.

[143] Feng L., Shi Z., Chen X. (2017). Enhancer of polycomb coordinates multiple signaling pathways to promote both cyst and germline stem cell differentiation in the *Drosophila* adult testis. PLoS Genet..

[144] Feng L., Shi Z., Xie J., Ma B., Chen X. (2018). Enhancer of polycomb maintains germline activity and genome integrity in *Drosophila* testis. Cell Death Differ..

[145] Tarayrah L., Herz H.M., Shilatifard A., Chen X. (2013). Histone demethylase dUTX antagonizes JAK-STAT signaling to maintain proper gene expression and architecture of the *Drosophila* testis niche. Development.

[146] Monahan A.J., Starz-Gaiano M. (2016). Apontic regulates somatic stem cell numbers in *Drosophila* testes. BMC Dev. Biol..

[147] Terry N.A., Tulina N., Matunis E., DiNardo S. (2006). Novel regulators revealed by profiling *Drosophila* testis stem cells within their niche. Dev. Biol..

[148] Feng Y., Spezia M., Huang S., Yuan C., Zeng Z., Zhang L., Ji X., Liu W., Huang B., Luo W. (2018). Breast cancer development and progression: Risk factors, cancer stem cells, signaling pathways, genomics, and molecular pathogenesis. Genes Dis..

[149] Yamashita H., Iwase H., Toyama T., Fujii Y. (2003). Naturally occurring dominant-negative Stat5 suppresses transcriptional activity of estrogen receptors and induces apoptosis in T47D breast cancer cells. Oncogene.

[150] Sp N., Darvin P., Yoo Y.B., Joung Y.H., Kang D.Y., Kim D.N., Hwang T.S., Kim S.Y., Kim W.S., Lee H.K. (2015). The combination of methylsulfonylmethane and tamoxifen inhibits the Jak2/STAT5b pathway and synergistically inhibits tumor growth and metastasis in ER-positive breast cancer xenografts. BMC Cancer.

[151] Chang R., Song L., Xu Y., Wu Y., Dai C., Wang X., Sun X., Hou Y., Li W., Zhan X. (2018). Loss of Wwox drives metastasis in triple-negative breast cancer by JAK2/STAT3 axis. Nat. Commun..

[152] Aysola K., Desai A., Welch C., Xu J., Qin Y., Reddy V., Matthews R., Owens C., Okoli J., Beech D.J. (2013). Triple Negative Breast Cancer—An Overview. Hered. Genet..

[153] Balko J.M., Schwarz L.J., Cook R.S., Estrada M.V., Giltnane J.M., Sanders M.E., Snchez V., Dean P.T., Wang K., Combs S.E. (2016). Triple negative breast cancers with amplification of JAK2 at the 9p24 loci demonstrate JAK2-specific dependence. Sci. Transl. Med..

[154] Chang Q., Bournazou E., Sansone P., Berishaj M., Gao S.P., Daly L., Wels J., Theilen T., Granitto S., Zhang X. (2013). The IL-6/JAK/Stat3 Feed-Forward Loop Drives Tumorigenesis and Metastasis. Neoplasia.

[155] Krebs D.L., Hilton D.J. (2000). SOCS: Physiological suppressors of cytokine signaling. J. Cell Sci..

[156] Qian Q., Lv Y., Li P. (2018). SOCS1 is associated with clinical progression and acts as an oncogenic role in triple-negative breast cancer. IUBMB Life.

[157] Kim M.S., Jeong J., Seo J., Kim H.-S., Kim S.-J., Jin W. (2016). Dysregulated JAK2 expression by TrkC promotes metastasis potential, and EMT program of metastatic breast cancer. Sci. Rep..

[158] Yu J.-M., Sun W., Hua F., Xie J., Lin H., Zhou D.-D., Hu Z.-W. (2015). BCL6 induces EMT by promoting the ZEB1-mediated transcription repression of E-cadherin in breast cancer cells. Cancer Lett..

[159] Tran T.H., Utama F.E., Lin J., Yang N., Sjolund A.B., Ryder A., Johnson K.J., Neilson L.M., Liu C., Brill K.L. (2010). Prolactin Inhibits Expression of the Proto-oncogene BCL6 in Breast Cancer through a Stat5a Dependent Mechanism. Cancer Res..

[160] Johnson K.J., Peck A.R., Liu C., Tran T.H., Utama F.E., Sjolund A.B., Schaber J.D., Witkiewicz A.K., Rui H. (2010). PTP1B Suppresses Prolactin Activation of Stat5 in Breast Cancer Cells. Am. J. Pathol..

[161] Liao S.-C., Li J.-X., Yu L., Sun S.-R. (2017). Protein tyrosine phosphatase 1B expression contributes to the development of breast cancer. J. Zhejiang Univ. Sci. B.

[162] Dadakhujaev S., Salazar-Arcila C., Netherton S.J., Chandhoke A.S., Singla A.K., Jirik F.R., Bonni S. (2014). A novel role for the SUMO E3 ligase PIAS1 in cancer metastasis. Oncoscience.

[163] Hoang D.T., Gu L., Liao Z., Talati P.G., Shen F., Koptyra M., Tan S.-H., Ellsworth E., Gupta S., Montie H. (2015). Inhibition of Stat5a/b enhances proteasomal degradation of androgen receptor liganded by antiandrogens in prostate cancer. Mol. Cancer Ther..

[164] Gu L., Vogiatzi P., Puhr M., Dagvadorj A., Lutz J., Ryder A., Addya S., Fortina P., Cooper C., Leiby B. (2010). Stat5 promotes metastatic behavior of human prostate cancer cells in vitro and in vivo. Endocr.-Relat. Cancer.

[165] Santer F.R., Malinowska K., Culig Z., Cavarretta I.T. (2010). Interleukin-6 trans-signalling differentially regulates proliferation, migration, adhesion and maspin expression in human prostate cancer cells. Endocr.-Relat. Cancer.

[166] Talati P.G., Gu L., Ellsworth E.M., Girondo M.A., Trerotola M., Hoang D.T., Leiby B., Dagvadorj A., McCue P.A., Lallas C.D. (2015). Jak2-Stat5a/b Signaling Induces Epithelial-to-Mesenchymal Transition and Stem-Like Cell Properties in Prostate Cancer. Am. J. Pathol..

[167] Gu L., Liao Z., Hoang D., Dagvadorj A., Gupta S., Blackmon S., Ellsworth E., Talati P., Leiby B., Zinda M. (2013). Pharmacological Inhibition of Jak2-Stat5 Signaling by Jak2 Inhibitor Azd1480 Potently Suppresses Growth of Both Primary and Castrate-Resistant Prostate Cancer. Clin. Cancer Res..

[168] Da Silva H.B., Amaral E., Nolasco E.L., deVicto N., Atique R., Jank C.C., Anschau V., Zerbini L.F., Correa R.G. (2013). Dissecting Major Signaling Pathways throughout the Development of Prostate Cancer. Prostate Cancer.

[169] Villalobos-Hernandez A., Bobbala D., Kandhi R., Khan M.G.M., Mayhue M., Dubois C.M., Ferbeyre G., Saucier C., Ramanathan S., Ilangumaran S. (2016). SOCS1 inhibits migration and invasion of prostate cancer cells, attenuates tumor growth and modulates the tumor stroma. Prostate Cancer Prostatic Dis..

[170] Shariat S.F., Chromecki T.F., Hoefer J., Barbieri C.E., Scherr D.S., Karakiewicz P.I., Roehrborn C.G., Montorsi F., Culig Z., Cavarretta I.T. (2011). Soluble gp130 Regulates Prostate Cancer Invasion and Progression in an Interleukin-6 Dependent and Independent Manner. J. Urol..

[171] Lessard L., Labb (2012). PTP1B Is an Androgen Receptor–Regulated Phosphatase That Promotes the Progression of Prostate Cancer. Cancer Res..

[172] Puhr M., Hoefer J., Eigentler A., Dietrich D., van Leenders G., Uhl B., Hoogland M., Handle F., Schlick B., Neuwirt H. (2016). PIAS1 is a determinant of poor survival and acts as a positive feedback regulator of AR signaling through enhanced AR stabilization in prostate cancer. Oncogene.

[173] Duhart J.C., Parsons T.T., Raftery L.A. (2017). The repertoire of epithelial morphogenesis on display: Progressive elaboration of *Drosophila* egg structure. Mech. Dev..

[174] Denef N., Schupbach T. (2003). Patterning: JAK-STAT signalling in the *Drosophila* follicular epithelium. Curr. Biol..

[175] Van de Bor V., Zimniak G., Cerezo D., Schaub S., Noselli S. (2011). Asymmetric localisation of cytokine mRNA is essential for JAK/STAT activation during cell invasiveness. Development.

[176] Borensztejn A., Boissoneau E., Fernandez G., Agnes F., Pret A.M. (2013). JAK/STAT autocontrol of ligand-producing cell number through apoptosis. Development.

[177] Xi R., McGregor J.R., Harrison D.A. (2003). A gradient of JAK pathway activity patterns the anterior-posterior axis of the follicular epithelium. Dev. Cell.

[178] Starz-Gaiano M., Melani M., Wang X., Meinhardt H., Montell D.J. (2008). Feedback inhibition of Jak/STAT signaling by apontic is required to limit an invasive cell population. Dev. Cell.

[179] Saadin A., Starz-Gaiano M. (2016). Circuitous Genetic Regulation Governs a Straightforward Cell Migration. Trends Genet..

[180] Naora H., Montell D.J. (2005). Ovarian cancer metastasis: Integrating insights from disparate model organisms. Nat. Rev. Cancer.

[181] Yoshida H., Cheng W., Hung J., Montell D., Geisbrecht E., Rosen D., Liu J., Naora H. (2004). Lessons from border cell migration in the *Drosophila* ovary: A role for myosin VI in dissemination of human ovarian cancer. Proc. Natl. Acad. Sci. USA.

[182] Stuelten C.H., Parent C.A., Montell D.J. (2018). Cell motility in cancer invasion and metastasis: Insights from simple model organisms. Nat. Rev. Cancer.

[183] Pinheiro E.M., Montell D.J. (2004). Requirement for Par-6 and Bazooka in *Drosophila* border cell migration. Development.

[184] Wang H., Qiu Z., Xu Z., Chen S.J., Luo J., Wang X., Chen J. (2018). aPKC is a key polarity determinant in coordinating the function of three distinct cell polarities during collective migration. Development.

[185] Sotillos S., Krahn M., Espinosa-Vazquez J.M., Hombria J.C. (2013). Src kinases mediate the interaction of the apical determinant Bazooka/PAR3 with STAT92E and increase signalling efficiency in *Drosophila* ectodermal cells. Development.

[186] Niewiadomska P., Godt D., Tepass U. (1999). DE-Cadherin is required for intercellular motility during *Drosophila* oogenesis. J. Cell Biol..

[187] Cai D., Chen S.C., Prasad M., He L., Wang X., Choesmel-Cadamuro V., Sawyer J.K., Danuser G., Montell D.J. (2014). Mechanical feedback through E-cadherin promotes direction sensing during collective cell migration. Cell.

[188] Friedl P., Gilmour D. (2009). Collective cell migration in morphogenesis, regeneration and cancer. Nat Rev Mol. Cell. Biol..

[189] Haeger A., Wolf K., Zegers M.M., Friedl P. (2015). Collective cell migration: Guidance principles and hierarchies. Trends Cell Biol..

[190] Hegerfeldt Y., Tusch M., Brocker E.B., Friedl P. (2002). Collective cell movement in primary melanoma explants: Plasticity of cell-cell interaction, beta1-integrin function, and migration strategies. Cancer Res..

[191] Khalil A.A., Ilina O., Gritsenko P.G., Bult P., Span P.N., Friedl P. (2017). Collective invasion in ductal and lobular breast cancer associates with distant metastasis. Clin. Exp. Metastasis.

[192] Yoon W.H., Meinhardt H., Montell D. (2011). miRNA-mediated feedback inhibition of JAK/STAT morphogen signalling establishes a cell fate threshold. Nat. Cell Biol..

[193] Starz-Gaiano M., Melani M., Meinhardt H., Montell D. (2009). Interpretation of the UPD/JAK/STAT morphogen gradient in *Drosophila* follicle cells. Cell Cycle.

[194] Maimon I., Popliker M., Gilboa L. (2014). Without children is required for Stat-mediated zfh1 transcription and for germline stem cell differentiation. Development.

[195] Saadin A., Starz-Gaiano M. (2018). Cytokine exocytosis and JAK/STAT activation in the *Drosophila* ovary requires the vesicle trafficking regulator α-Snap *J*. Cell Sci..

[196] Sengupta S., Michener C.M., Escobar P., Belinson J., Ganapathi R. (2008). Ovarian cancer immuno-reactive antigen domain containing 1 (OCIAD1), a key player in ovarian cancer cell adhesion. Gynecol. Oncol..

[197] Kulkarni V., Khadilkar R.J., Magadi S.S., Inamdar M.S. (2011). Asrij maintains the stem cell niche and controls differentiation during *Drosophila* lymph gland hematopoiesis. PLoS ONE.

[198] Sinha S., Bheemsetty V.A., Inamdar M.S. (2018). A double helical motif in OCIAD2 is essential for its localization, interactions and STAT3 activation. Sci. Rep..

[199] De Semir D., Nosrati M., Bezrookove V., Dar A.A., Federman S., Bienvenu G., Venna S., Rangel J., Climent J., Tamguney T.M. (2012). Pleckstrin homology domain-interacting protein (PHIP) as a marker and mediator of melanoma metastasis. Proc. Natl. Acad. Sci. USA.

[200] Huang X., Zhang H., Guo X., Zhu Z., Cai H., Kong X. (2018). Insulin-like growth factor 2 mRNA-binding protein 1 (IGF2BP1) in cancer. J. Hematol. Oncol..

[201] Su Y., Xiong J., Hu J., Wei X., Zhang X., Rao L. (2016). MicroRNA-140-5p targets insulin like growth factor 2 mRNA binding protein 1 (IGF2BP1) to suppress cervical cancer growth and metastasis. Oncotarget.

[202] Qu Y., Pan S., Kang M., Dong R., Zhao J. (2016). MicroRNA-150 functions as a tumor suppressor in osteosarcoma by targeting IGF2BP1. Tumor Biol..

[203] Luo Y., Sun R., Zhang J., Sun T., Liu X., Yang B. (2015). miR-506 inhibits the proliferation and invasion by targeting IGF2BP1 in glioblastoma. Am. J. Transl. Res..

[204] Zhou X., Zhang C.Z., Lu S.X., Chen G.G., Li L.Z., Liu L.L., Yi C., Fu J., Hu W., Wen J.M. (2014). miR-625 suppresses tumour migration and invasion by targeting IGF2BP1 in hepatocellular carcinoma. Oncogene.

[205] Kato T., Hayama S., Yamabuki T., Ishikawa N., Miyamoto M., Ito T., Tsuchiya E., Kondo S., Nakamura Y., Daigo Y. (2007). Increased Expression of Insulin-like Growth Factor-II Messenger RNA–Binding Protein 1 Is Associated with Tumor Progression in Patients with Lung Cancer. Clin. Cancer Res..

[206] Toledano H., D’Alterio C., Czech B., Levine E., Jones D.L. (2012). The let-7-Imp axis regulates ageing of the *Drosophila* testis stem-cell niche. Nature.

[207] Hwang J.A., Song J.S., Yu D.Y., Kim H.R., Park H.J., Park Y.S., Kim W.S., Choi C.M. (2015). Peroxiredoxin 4 as an independent prognostic marker for survival in patients with early-stage lung squamous cell carcinoma. Int. J. Clin. Exp. Pathol..

[208] Basu A., Banerjee H., Rojas H., Martinez S.R., Roy S., Jia Z., Lilly M.B.a. (2011). Differential Expression of Peroxiredoxins in Prostate Cancer: Consistent Upregulation of PRDX3 and PRDX4. Prostate.

[209] Kim T.H., Song J.a. (2012). Suppression of Peroxiredoxin 4 in Glioblastoma Cells Increases Apoptosis and Reduces Tumor Growth. PLoS ONE.

[210] Radyuk S.N., Klichko V.I., Michalak K., Orr W.C. (2013). The effect of peroxiredoxin 4 on fly physiology is a complex interplay of antioxidant and signaling functions. FASEB J..

[211] Wang Y.-H., Wu W.-J., Wang W.-J., Huang H.-Y., Li W.-M., Yeh B.-W., Wu T.-F., Shiue Y.-L., Sheu J.J.-C., Wang J.-M. (2015). CEBPD amplification and overexpression in urothelial carcinoma: A driver of tumor metastasis indicating adverse prognosis. Oncotarget.

[212] Grinberg-Rashi H., Ofek E., Perelman M., Skarda J., Yaron P., Hajdch M., Jacob-Hirsch J., Amariglio N., Krupsky M., Simansky D.A. (2009). The Expression of Three Genes in Primary Non–Small Cell Lung Cancer Is Associated with Metastatic Spread to the Brain. Clin. Cancer Res..

[213] Dar A.A., Nosrati M., Bezrookove V., de Semir D., Majid S., Thummala S., Sun V., Tong S., Leong S.P.L., Minor D. (2015). The Role of BPTF in Melanoma Progression and in Response to BRAF-Targeted Therapy. JNCI J. Natl. Cancer Inst..

[214] Dar A.A., Majid S., Bezrookove V., Phan B., Ursu S., Nosrati M.a. (2016). BPTF transduces MITF-driven prosurvival signals in melanoma cells. Proc. Natl. Acad. Sci. USA.

[215] Dai M., Lu J.-J., Guo W., Yu W., Wang Q., Tang R., Tang Z., Xiao Y., Li Z., Sun W. (2015). BPTF promotes tumor growth and predicts poor prognosis in lung adenocarcinomas. Oncotarget.

[216] Xiao S., Liu L., Fang M., Zhou X., Peng X., Long J., Lu X. (2015). BPTF Associated with EMT Indicates Negative Prognosis in Patients with Hepatocellular Carcinoma. Dig. Dis. Sci..

[217] Xiao S., Liu L., Lu X., Long J., Zhou X., Fan M. (2015). The prognostic significance of bromodomain PHD-finger transcription factor in colorectal carcinoma and association with vimentin and E-cadherin. J. Cancer Res. Clin. Oncol..

[218] Latonen L., Leinonen K.A., Grnlund T., Vessella R.L., Tammela T.L.J., Saramki O.R., Visakorpi T. (2016). Amplification of the 9p13.3 chromosomal region in prostate cancer. Genes Chromosom. Cancer.

[219] Bai D.-S., Wu C., Yang L.-X., Zhang C., Zhang P.-F., He Y.-Z., Cai J.-B., Song Z.-J., Dong Z.-R., Huang X.-Y. (2016). UBAP2 negatively regulates the invasion of hepatocellular carcinoma cell by ubiquitinating and degradating Annexin A2. Oncotarget.

[220] Baumgartner R., Stocker H., Hafen E. (2013). The RNA-binding proteins FMR1, rasputin and caprin act together with the UBA protein lingerer to restrict tissue growth in *Drosophila* melanogaster. PLoS Genet..

[221] Gunsalus K.T.W., Wagoner M.P., Meyer K., Potter W.B., Schoenike B., Kim S., Alexander C.M., Friedl A., Roopra A. (2012). Induction of the RNA Regulator LIN28A is Required for the Growth and Pathogenesis of RESTless Breast Tumors. Cancer Res..

[222] Liang H., Studach L., Hullinger R.L., Xie J., Andrisani O.M. (2014). Down-regulation of RE-1 Silencing Transcription Factor (REST) in advanced prostate cancer by hypoxia-induced miR-106b\25. Exp. Cell Res..

[223] Kugler S.J., Gehring E.M., Wallkamm V., Kruger V., Nagel A.C. (2011). The Putzig-NURF nucleosome remodeling complex is required for ecdysone receptor signaling and innate immunity in *Drosophila* melanogaster. Genetics.

[224] Gao T., Zheng S., Li Q., Ran P., Sun L., Yuan Y., Xiao C. (2015). Aberrant hypomethylation and overexpression of the eyes absent homologue 2 suppresses tumor cell growth of human lung adenocarcinoma cells. Oncol. Rep..

[225] Liang Y., Xu X., Wang T., Li Y., You W., Fu J., Liu Y., Jin S., Ji Q., Zhao W. (2017). The EGFR/miR-338-3p/EYA2 axis controls breast tumor growth and lung metastasis. Cell Death Dis..

[226] Vincent A., Hong S.-M., Hu C., Omura N., Young A., Kim H., Yu J., Knight S., Ayars M., Griffith M.a. (2014). Epigenetic silencing of EYA2 in pancreatic adenocarcinomas promotes tumor growth. Oncotarget.

[227] Copf T., Goguel V., Lampin-Saint-Amaux A., Scaplehorn N., Preat T. (2011). Cytokine signaling through the JAK/STAT pathway is required for long-term memory in *Drosophila*. Proc. Natl. Acad. Sci. USA.

[228] Johansen K.A., Iwaki D.D., Lengyel J.A. (2003). Localized JAK/STAT signaling is required for oriented cell rearrangement in a tubular epithelium. Development.

[229] Li J., Li W., Calhoun H.C., Xia F., Gao F.B., Li W.X. (2003). Patterns and functions of STAT activation during *Drosophila* embryogenesis. Mech. Dev..

[230] Baksa K., Parke T., Dobens L.L., Dearolf C.R. (2002). The *Drosophila* STAT protein, stat92E, regulates follicle cell differentiation during oogenesis. Dev. Biol..

[231] Sheng X.R., Posenau T., Gumulak-Smith J.J., Matunis E., Van Doren M., Wawersik M. (2009). Jak-STAT regulation of male germline stem cell establishment during *Drosophila* embryogenesis. Dev. Biol..

[232] Li J., Xia F., Li W.X. (2003). Coactivation of STAT and Ras is required for germ cell proliferation and invasive migration in *Drosophila*. Dev. Cell.

[233] Brown S., Zeidler M.P., Hombria J.E. (2006). JAK/STAT signalling in *Drosophila* controls cell motility during germ cell migration. Dev. Dyn..

[234] Cao S., Wang C., Zheng Q., Qiao Y., Xu K., Jiang T., Wu A. (2011). STAT5 regulates glioma cell invasion by pathways dependent and independent of STAT5 DNA binding. Neurosci. Lett..

[235] Wolf Monika, Hoos A., Bauer J., Boettcher S., Knust M., Weber A., Simonavicius N., Schneider C., Lang M., Strzl M. (2012). Endothelial CCR2 Signaling Induced by Colon Carcinoma Cells Enables Extravasation via the JAK2-Stat5 and p38MAPK Pathway. Cancer Cell.

[236] Xiong H., Su W.-Y., Liang Q.-C., Zhang Z.-G., Chen H.-M., Du W., Chen Y.-X., Fang J.-Y. (2009). Inhibition of STAT5 induces G1 cell cycle arrest and reduces tumor cell invasion in human colorectal cancer cells. Lab. Investig..

[237] Klupp F., Diers J., Kahlert C., Neumann L., Halama N., Franz C., Schmidt T., Lasitschka F., Warth A., Weitz J. (2015). Expressional STAT3/STAT5 Ratio is an Independent Prognostic Marker in Colon Carcinoma. Ann. Surg. Oncol..

[238] Wellbrock C., Weisser C., Hassel J.C., Fischer P., Becker J., Vetter C.S., Behrmann I., Kortylewski M., Heinrich P.C., Schartl M. (2005). STAT5 Contributes to Interferon Resistance of Melanoma Cells. Curr. Biol..

[239] Moser C., Ruemmele P., Gehmert S., Schenk H., Kreutz M.P., Mycielska M.E., Hackl C., Kroemer A., Schnitzbauer A.A., Stoeltzing O. (2012). STAT5b as Molecular Target in Pancreatic Cancer—Inhibition of Tumor Growth, Angiogenesis, and Metastases. Neoplasia.

[240] Luo H., Asha H., Kockel L., Parke T., Mlodzik M., Dearolf C.R. (1999). The *Drosophila* Jak kinase hopscotch is required for multiple developmental processes in the eye. Dev. Biol..

[241] Liu Y.H., Jakobsen J.S., Valentin G., Amarantos I., Gilmour D.T., Furlong E.E. (2009). A systematic analysis of Tinman function reveals Eya and JAK-STAT signaling as essential regulators of muscle development. Dev. Cell.

[242] McGregor J.R., Xi R., Harrison D.A. (2002). JAK signaling is somatically required for follicle cell differentiation in *Drosophila*. Development.

[243] Yun H.M., Park K.R., Quang T.H., Oh H., Hong J.T., Kim Y.C., Kim E.C. (2017). 4-parvifuran inhibits metastatic and invasive actions through the JAK2/STAT3 pathway in osteosarcoma cells. Arch. Pharm. Res..

[244] Luo C.L., Liu Y.Q., Wang P., Song C.H., Wang K.J., Dai L.P., Zhang J.Y., Ye H. (2016). The effect of quercetin nanoparticle on cervical cancer progression by inducing apoptosis, autophagy and anti-proliferation via JAK2 suppression. Biomed. Pharmacother..

[245] Liu X., Ji Q., Ye N., Sui H., Zhou L., Zhu H., Fan Z., Cai J., Li Q. (2015). Berberine Inhibits Invasion and Metastasis of Colorectal Cancer Cells via COX-2/PGE(2) Mediated JAK2/STAT3 Signaling Pathway. PLoS ONE.

[246] Shin D.S., Zaretsky J.M., Escuin-Ordinas H., Garcia-Diaz A., Hu-Lieskovan S., Kalbasi A., Grasso C.S., Hugo W., Sandoval S., Torrejon D.Y. (2017). Primary resistance to PD-1 blockade mediated by JAK1/2 mutations. Cancer Discov..

[247] Das S., Rachagani S., Torres-Gonzalez M.P., Lakshmanan I., Majhi P.D., Smith L.M., Wagner K.U., Batra S.K. (2015). Carboxyl-terminal domain of MUC16 imparts tumorigenic and metastatic functions through nuclear translocation of JAK2 to pancreatic cancer cells. Oncotarget.

[248] Tsai Y.C., Sun Y.H. (2004). Long-range effect of upd, a ligand for Jak/STAT pathway, on cell cycle in *Drosophila* eye development. Genesis.

[249] Medioni C., Noselli S. (2005). Dynamics of the basement membrane in invasive epithelial clusters in *Drosophila*. Development.

[250] Tu B., Du L., Fan Q.M., Tang Z., Tang T.T. (2012). STAT3 activation by IL-6 from mesenchymal stem cells promotes the proliferation and metastasis of osteosarcoma. Cancer Lett..

[251] Schneider M.R., Hoeflich A., Fischer J.R., Wolf E., Sordat B., Lahm H. (2000). Interleukin-6 stimulates clonogenic growth of primary and metastatic human colon carcinoma cells. Cancer Lett..

[252] Zeng J., Tang Z.-H., Liu S., Guo S.-S. (2017). Clinicopathological significance of overexpression of interleukin-6 in colorectal cancer. World J. Gastroenterol..

[253] Zhang X., Hu F., Li G., Li G., Yang X., Liu L., Zhang R., Zhang B., Feng Y. (2018). Human colorectal cancer-derived mesenchymal stem cells promote colorectal cancer progression through IL-6/JAK2/STAT3 signaling. Cell Death Dis..

[254] Pu X.Y., Zheng D.F., Shen A., Gu H.T., Wei X.F., Mou T., Zhang J.B., Liu R. (2018). IL-37b suppresses epithelial mesenchymal transition in hepatocellular carcinoma by inhibiting IL-6/STAT3 signaling. Hepatobiliary Pancreat Dis. Int..

[255] Zou M., Zhang X., Xu C. (2016). IL6-induced metastasis modulators p-STAT3, MMP-2 and MMP-9 are targets of 3,3′-diindolylmethane in ovarian cancer cells. Cell. Oncol..

[256] Grunwald B., Vandooren J., Gerg M., Ahomaa K., Hunger A., Berchtold S., Akbareian S., Schaten S., Knolle P., Edwards D.R. (2016). Systemic Ablation of MMP-9 Triggers Invasive Growth and Metastasis of Pancreatic Cancer via Deregulation of IL6 Expression in the Bone Marrow. Mol. Cancer Res..

[257] Lacreusette A., Nguyen J.M., Pandolfino M.C., Khammari A., Dreno B., Jacques Y., Godard A., Blanchard F. (2006). Loss of oncostatin M receptor $\beta$ in metastatic melanoma cells. Oncogene.

[258] Kuhnlein R.P., Chen C.K., Schuh R. (1998). A transcription unit at the ken and barbie gene locus encodes a novel *Drosophila* zinc finger protein. Mech. Dev..

[259] Lukacsovich T., Yuge K., Awano W., Asztalos Z., Kondo S., Juni N., Yamamoto D. (2003). The ken and barbie gene encoding a putative transcription factor with a BTB domain and three zinc finger motifs functions in terminalia development of *Drosophila*. Arch. Insect. Biochem. Physiol..

[260] Chen Q., Li Y., Li Z., Zhao Q., Fan L. (2014). Overexpression of PTP1B in human colorectal cancer and its association with tumor progression and prognosis. J. Mol. Histol..

[261] Wang X.-M., Shang L., Zhang Y., Hao J.-J., Shi F., Luo W., Zhang T.-T., Wang B.-S., Yang Y., Liu Z.-H. (2013). PTP1B Contributes to Calreticulin-Induced Metastatic Phenotypes in Esophageal Squamous Cell Carcinoma. Mol. Cancer Res..

[262] Julien S.G., Dubé N., Read M., Penney J., Paquet M., Han Y., Kennedy B.P., Muller W.J., Tremblay M.L. (2007). Protein tyrosine phosphatase 1B deficiency or inhibition delays ErbB2-induced mammary tumorigenesis and protects from lung metastasis. Nat. Genet..

[263] Liu J., Luan W., Zhang Y., Gu J., Shi Y., Yang Y., Feng Z., Qi F. (2018). HDAC6 interacts with PTPN1 to enhance melanoma cells progression. Biochem. Biophys. Res. Commun..

[264] Fan G., Lin G., Lucito R., Tonks N.K. (2013). Protein-tyrosine phosphatase 1B antagonized signaling by insulin-like growth factor-1 receptor and kinase BRK/PTK6 in ovarian cancer cells. J. Biol. Chem..

[265] Chen P., Zhao D., Sun Y., Huang L., Zhang S., Yuan Y. (2012). Protein inhibitor of activated STAT-1 is downregulated in gastric cancer tissue and involved in cell metastasis. Oncol. Rep..

[266] David M., Naudin C., Letourneur M., Polrot M., Renoir J.-M., Lazar V., Dessen P., Roche S., Bertoglio J., Pierre J. (2014). Suppressor of cytokine signaling 1 modulates invasion and metastatic potential of colorectal cancer cells. Mol. Oncol..

[267] Gui Y., Khan M.G.M., Bobbala D., Dubois C., Ramanathan S., Saucier C., Ilangumaran S. (2017). Attenuation of MET-mediated migration and invasion in hepatocellular carcinoma cells by SOCS1. World J. Gastroenterol..

[268] Scutti J.A.B., Matsuo A.L., Pereira F.V., Massaoka M.H., Figueiredo C.R., Moreira D.F., Belizrio J.E., Travassos L.R. (2011). Role of SOCS-1 Gene on Melanoma Cell Growth and Tumor Development. Transl. Oncol..

[269] Su M., Qin B., Liu F., Chen Y., Zhang R. (2018). miR-885-5p upregulation promotes colorectal cancer cell proliferation and migration by targeting suppressor of cytokine signaling. Oncol. Lett..

[270] Sanchez-Mejias A., Kwon J., Chew X.H., Siemens A., Sohn H.S., Jing G., Zhang B., Yang H., Tay Y. (2018). A novel SOCS5/miR-18/miR-25 axis promotes tumorigenesis in liver cancer. Int. J. Cancer.

[271] Kwon S.Y., Xiao H., Wu C., Badenhorst P. (2009). Alternative splicing of NURF301 generates distinct NURF chromatin remodeling complexes with altered modified histone binding specificities. PLoS Genet..

[272] Costa A., Pazman C., Sinsimer K.S., Wong L.C., McLeod I., Yates J., Haynes S., Schedl P. (2013). Rasputin functions as a positive regulator of orb in *Drosophila* oogenesis. PLoS ONE.

[273] Cammarato A., Ahrens C.H., Alayari N.N., Qeli E., Rucker J., Reedy M.C., Zmasek C.M., Gucek M., Cole R.N., Van Eyk J.E. (2011). A mighty small heart: The cardiac proteome of adult *Drosophila* melanogaster. PLoS ONE.

[274] Bonini N.M., Leiserson W.M., Benzer S. (1993). The eyes absent gene: Genetic control of cell survival and differentiation in the developing *Drosophila* eye. Cell.

[275] Rayapureddi J.P., Kattamuri C., Steinmetz B.D., Frankfort B.J., Ostrin E.J., Mardon G., Hegde R.S. (2003). Eyes absent represents a class of protein tyrosine phosphatases. Nature.

[276] Xiong W., Dabbouseh N.M., Rebay I. (2009). Interactions with the Abelson tyrosine kinase reveal compartmentalization of eyes absent function between nucleus and cytoplasm. Dev. Cell.

[277] Vining M.S., Bradley P.L., Comeaux C.A., Andrew D.J. (2005). Organ positioning in *Drosophila* requires complex tissue-tissue interactions. Dev. Biol..

[278] Bai J., Montell D. (2002). Eyes absent, a key repressor of polar cell fate during *Drosophila* oogenesis. Development.

[279] Fabrizio J.J., Boyle M., DiNardo S. (2003). A somatic role for eyes absent (eya) and sine oculis (so) in *Drosophila* spermatocyte development. Dev. Biol..

[280] Wen Z., Liang C., Pan Q., Wang Y. (2017). Eya2 overexpression promotes the invasion of human astrocytoma through the regulation of ERK/MMP9 signaling. Int. J. Mol. Med..

[281] Farabaugh S.M., Micalizzi D.S., Jedlicka P., Zhao R., Ford H.L. (2012). Eya2 Is Required to Mediate the Pro-Metastatic Functions of Six1 Via the Induction of TGF-β Signaling, Epithelial-Mesenchymal Transition, and Cancer Stem Cell Properties. Oncogene.

[282] Krueger A.B., Drasin D.J., Lea W.A., Patrick A.N., Patnaik S., Backos D.S., Matheson C.J., Hu X., Barnaeva E., Holliday M.J. (2014). Allosteric inhibitors of the Eya2 phosphatase are selective and inhibit Eya2-mediated cell migration. J. Biol. Chem..

[283] Aradska J., Bulat T., Sialana F.J., Birner-Gruenberger R., Erich B., Lubec G. (2015). Gel-free mass spectrometry analysis of *Drosophila* melanogaster heads. Proteomics.

[284] Chen W.Y., Shih H.T., Liu K.Y., Shih Z.S., Chen L.K., Tsai T.H., Chen M.J., Liu H., Tan B.C., Chen C.Y. (2015). Intellectual disability-associated dBRWD3 regulates gene expression through inhibition of HIRA/YEM-mediated chromatin deposition of histone H3.3. EMBO Rep..

[285] Ihry R.J., Bashirullah A. (2014). Genetic control of specificity to steroid-triggered responses in *Drosophila*. Genetics.

[286] Wasbrough E.R., Dorus S., Hester S., Howard-Murkin J., Lilley K., Wilkin E., Polpitiya A., Petritis K., Karr T.L. (2010). The *Drosophila* melanogaster sperm proteome-II (DmSP-II). J. Proteom..

[287] Bezrookove V.a. (2014). Prognostic Impact of PHIP Copy Number in Melanoma: Linkage to Ulceration. J. Investig. Dermatol..

[288] Munro T.P., Kwon S., Schnapp B.J., St Johnston D. (2006). A repeated IMP-binding motif controls oskar mRNA translation and anchoring independently of *Drosophila* melanogaster IMP. J. Cell Biol..

[289] Jiang T., Li M., Li Q., Guo Z., Sun X., Zhang X., Liu Y., Yao W., Xiao P. (2017). MicroRNA-98-5p Inhibits Cell Proliferation and Induces Cell Apoptosis in Hepatocellular Carcinoma via Targeting IGF2BP1. Oncol. Res..

[290] Yuan P., Meng L., Wang N. (2017). SOX12 upregulation is associated with metastasis of hepatocellular carcinoma and increases CDK4 and IGF2BP1 expression. Eur. Rev. Med. Pharmacol. Sci..

[291] Zhang J., Cheng J., Zeng Z., Wang Y., Li X., Xie Q., Jia J., Yan Y., Guo Z., Gao J. (2015). Comprehensive profiling of novel microRNA-9 targets and a tumor suppressor role of microRNA-9 via targeting IGF2BP1 in hepatocellular carcinoma. Oncotarget.

[292] Montell D.J., Rorth P., Spradling A.C. (1992). slow border cells, a locus required for a developmentally regulated cell migration during oogenesis, encodes *Drosophila* C/EBP. Cell.

[293] Min Y., Ghose S., Boelte K., Li J., Yang L., Lin P.C. (2011). C/EBP-β regulates VEGF-C autocrine signaling in lymphangiogenesis and metastasis of lung cancer through HIF-1α. Oncogene.

[294] Hsiao Y.-W., Li C.-F., Chi J.-Y., Tseng J.T., Chang Y., Hsu L.-J., Lee C.-H., Chang T.-H., Wang S.-M., Wang D.D.H. (2013). CCAAT/Enhancer Binding Protein β in Macrophages Contributes to Immunosuppression and Inhibits Phagocytosis in Nasopharyngeal Carcinoma. Sci. Signal..

[295] Vidal M., Larson D.E., Cagan R.L. (2006). Csk-deficient boundary cells are eliminated from normal *Drosophila* epithelia by exclusion, migration, and apoptosis. Dev. Cell.

[296] Read R.D., Bach E.A., Cagan R.L. (2004). *Drosophila* C-terminal Src kinase negatively regulates organ growth and cell proliferation through inhibition of the Src, Jun N-terminal kinase, and STAT pathways. Mol. Cell. Biol..

[297] Gerlach S.U., Eichenlaub T., Herranz H. (2018). Yorkie and JNK Control Tumorigenesis in *Drosophila* Cells with Cytokinesis Failure. Cell Rep..

[298] O’Reilly A.M., Ballew A.C., Miyazawa B., Stocker H., Hafen E., Simon M.A. (2006). Csk differentially regulates Src64 during distinct morphological events in *Drosophila* germ cells. Development.

[299] Rengifo-Cam W., Konishi A., Morishita N., Matsuoka H., Yamori T., Nada S., Okada M. (2004). Csk defines the ability of integrin-mediated cell adhesion and migration in human colon cancer cells: Implication for a potential role in cancer metastasis. Oncogene.

[300] Nakagawa T., Tanaka S., Suzuki H., Takayanagi H., Miyazaki T., Nakamura K., Tsuruo T. (2000). Overexpression of the csk gene suppresses tumor metastasis in vivo. Int. J. Cancer.

